# Initiation of stem cell differentiation involves cell cycle-dependent regulation of developmental genes by Cyclin D

**DOI:** 10.1101/gad.271452.115

**Published:** 2016-02-15

**Authors:** Siim Pauklin, Pedro Madrigal, Alessandro Bertero, Ludovic Vallier

**Affiliations:** 1 Wellcome Trust-Medical Research Council Cambridge Stem Cell Institute, Anne McLaren Laboratory for Regenerative Medicine, Department of Surgery, University of Cambridge, Madingley, Cambridge CB2 0SZ, United Kingdom;; 2 Wellcome Trust Sanger Institute, Hinxton, Cambridge CB10 1SA, United Kingdom

**Keywords:** hESCs, cell cycle, Cyclin D, differentiation, endoderm, neuroectoderm

## Abstract

Coordination of differentiation and cell cycle progression represents an essential process for embryonic development and adult tissue homeostasis. These mechanisms ultimately determine the quantities of specific cell types that are generated. Despite their importance, the precise molecular interplays between cell cycle machinery and master regulators of cell fate choice remain to be fully uncovered. Here, we demonstrate that cell cycle regulators Cyclin D1–3 control cell fate decisions in human pluripotent stem cells by recruiting transcriptional corepressors and coactivator complexes onto neuroectoderm, mesoderm, and endoderm genes. This activity results in blocking the core transcriptional network necessary for endoderm specification while promoting neuroectoderm factors. The genomic location of Cyclin Ds is determined by their interactions with the transcription factors SP1 and E2Fs, which result in the assembly of cell cycle-controlled transcriptional complexes. These results reveal how the cell cycle orchestrates transcriptional networks and epigenetic modifiers to instruct cell fate decisions.

## Abstract (precis)

Pauklin et al. show that cell cycle regulators Cyclin D1–3 control cell fate decisions in human pluripotent stem cells by recruiting transcriptional corepressors and coactivator complexes onto neuroectoderm, mesoderm, and endoderm genes. This activity results in blocking the core transcriptional network necessary for endoderm specification while promoting neuroectoderm factors.

Embryonic development, organogenesis, and tissue repair are controlled through the proliferation and differentiation of stem cells and progenitors. The importance of these mechanisms has been demonstrated in adult stem cells (Oshimori and Fuchs 2012) and also in pluripotent stem cells (Sela et al. 2012; Calder et al. 2013; Coronado et al. 2013; Singh et al. 2013, 2015). Human embryonic stem cells (hESCs) represent an advantageous model system for investigating the cross-talk between cell cycle and cell fate choice. hESCs can proliferate indefinitely while maintaining the capacity to differentiate into the three primary germ layers neuroectoderm, mesoderm, and endoderm in vitro. Furthermore, hESCs are characterized by a specific cell cycle profile with a short G1 phase, while their differentiation is associated with an increase in G1 phase length (Coronado et al. 2013). A recent study has shown that Cyclin D1–3/CDK4/6 complexes, which control G1 length and progression, also limit the transcriptional activity associated with Activin/Nodal signaling during the late G1 phase. This mechanism results in a cell cycle-dependent capacity of differentiation in which hESCs can only differentiate into endoderm in their early G1 phase and into neuroectoderm in their late G1 (Pauklin and Vallier 2013). Finally, S- and G2/M-related mechanisms appear to control the inhibition of pluripotency upon differentiation, suggesting the existence of differentiation checkpoints during cell cycle progression in stem cells (Gonzales et al. 2015). These reports collectively demonstrate that cell cycle regulators have a direct function in early events of differentiation by controlling the activity of extracellular signals. Interestingly, cell cycle regulators are also known to function at the chromatin level and control gene transcription (Sicinski et al. 2007; Pestell 2013). For instance, Cyclin D proteins have been found on DNA in mouse cancer cell lines but also in mouse retinal tissue (Bienvenu et al. 2010; Casimiro et al. 2012). However, the function of Cyclin D-mediated transcriptional mechanisms has never been studied in the context of stem cells and cell fate choice.

Here we demonstrate that the cell cycle machinery is directly involved in the transcriptional initiation of developmental genes controlling primary germ layer specification. Cyclin D1, which is highly expressed in late G1, recruits transcriptional corepressors to endoderm genes and coactivators to neuroectoderm genes, thereby blocking or promoting the induction of the corresponding germ layers. Thus, Cyclin D1 acts as a cell cycle-dependent transcriptional regulator, which can direct lineage specification of stem cells. These results show how cell cycle regulators can orchestrate cell fate decisions by organising transcriptional networks in human stem cells.

## Results

### *Cyclin D1* promotes neuroectoderm differentiation through chromatin-binding-dependent mechanisms that do not involve inhibition of *Smad2/3* by *CDK4/6* phosphorylation

**Figure 1.**
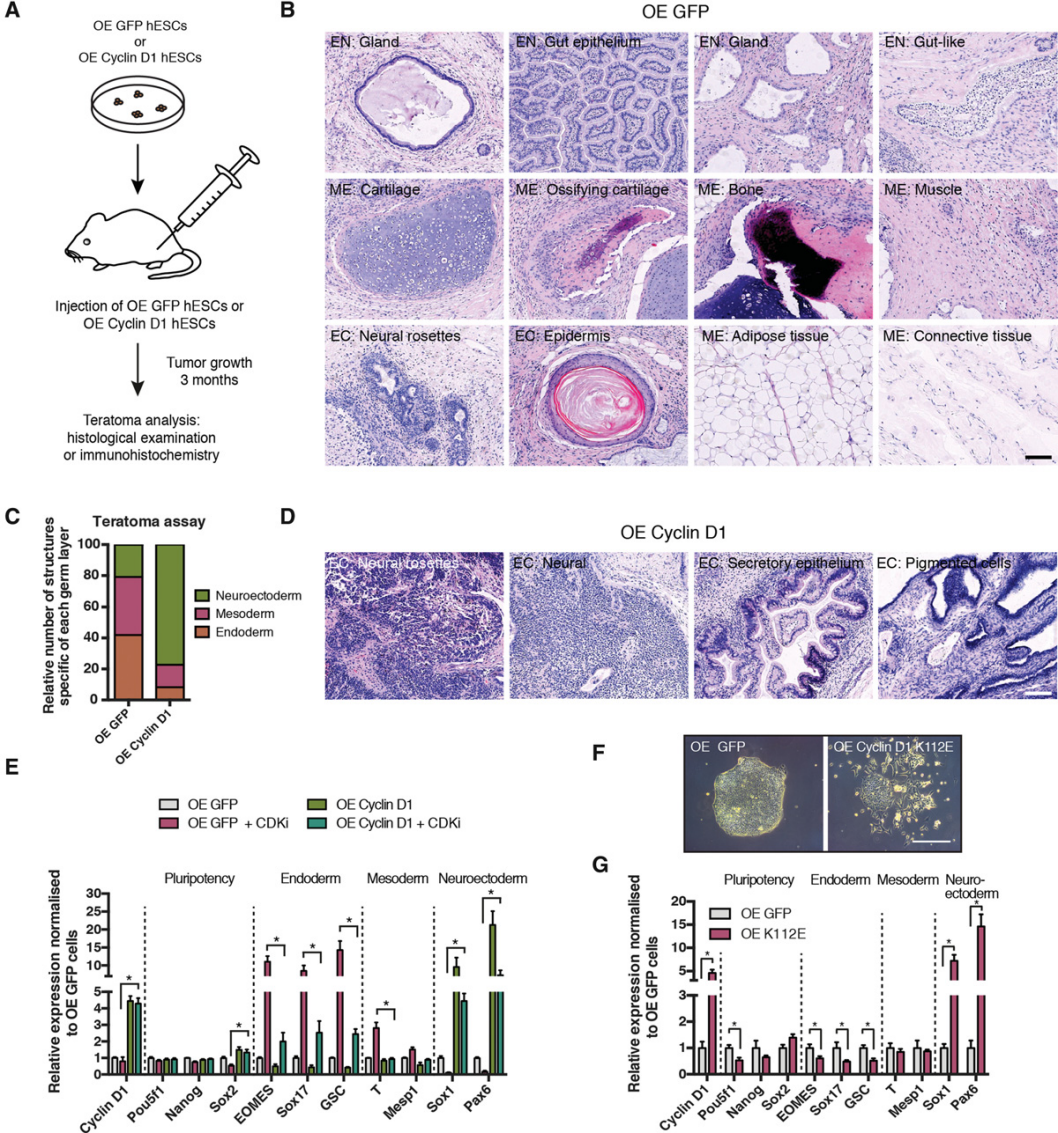
Cyclin D proteins can regulate cell fate decisions in hESCs independently of CDK4/6 activity. (*A*) Schematic overview of teratoma assay. (*B*) Histological sections of teratomas derived from GFP-overexpressing hESCs. (K) Kidney; (ME) mesoderm; (EN) endoderm; (NE) neuroectoderm. (*C*) Ratio of histological structures specific to each germ layer. *X*^2^= 152.5, Bonferroni corrected *P* < 6.6 × 10^−16^, χ^2^ test. (*D*) Histological sections of teratomas derived from Cyclin D1 overexpressing hESCs. (*E*) CDK4/6 inhibition does not fully abolish Cyclin D function. Significant differences calculated by two-way ANOVA are marked. (*F*) Morphology of a CycD1-K112E mutant overexpressing hESCs. Representative colonies of hESCs overexpressing GFP and CycD1-K112E. (*G*) The CycD1-K112E mutant, which is not able to activate CDK4/6, can block endoderm genes and induce neuroectoderm. Significant differences compared with overexpressing GFP and calculated by *t*-test are marked. All data are shown as mean ± SD. *n* = 3.

We recently showed that hESC differentiation is regulated by the cell cycle through mechanisms involving control of the Activin/Nodal signaling pathway via Smad2/3 phosphorylation by Cyclin D–CDK4/6 (Pauklin and Vallier 2013). We also observed that constitutive expression of Cyclin D1 and, to a lesser extent, Cyclin D2 and Cyclin D3 can rapidly increase the expression of neuronal markers independently of Smad2/3 inhibition. These results suggested that Cyclin Ds might prime the hESCs toward neuronal differentiation independently of Smad2/3–CDK4/6 cross-talk. To explore this hypothesis further, we decided to perform teratoma assays as an unbiased approach to evaluate pluripotency of hESCs overexpressing GFP or Cyclin D1 (Fig. 1A–D). Histological analyses of the resulting tumors were performed to define the proportion of germ layer derivatives generated. These analyses revealed that teratomas derived from control GFP-hESCs contained similar proportions of derivatives from the three germ layers, while Cyclin D1-hESC-derived teratomas contained 77% of neuroectodermal tissues (Fig. 1A–D; Supplemental Fig. S1A–C). In addition, statistical analyses showed that neuroectoderm was the main germ layer affected by Cyclin D1 overexpression (*X*^2^= 152.5, Bonferroni corrected; *P* < 6.6 × 10^−16^, χ^2^ test). Thus, Cyclin D1 appears to trigger differentiation of hESCs toward the neuroectodermal lineage independently of the surrounding environment. Next, we investigated whether Cyclin D1 could promote neuroectoderm specification in the absence of CDK4/6 activity by taking advantage of a highly specific CDK inhibitor, PD0332991 (Supplemental Fig. S1D; Fry et al. 2004). The addition of this small molecule in culture medium and thus the absence of Smad2/3 inhibition by CDK4/6 were not sufficient to block Cyclin D1 overexpression from inducing neuroectoderm and repressing endoderm differentiation, and this was confirmed by CDK4/6 knockdown (Fig. 1E; Supplemental Fig. S1E–H). Similar effects were obtained by overexpressing in hESCs a Cyclin D1 K112E mutant (CycD1-K112E) (Fig. 1F,G) that does not bind and activate CDK4/6 (Supplemental Fig. S1I; Baker et al. 2005). Considered together, these findings confirm that Cyclin D1 can direct cell fate decisions of hESCs independently of CDK4/6 activity.

### *Cyclin D1* inhibits endoderm differentiation through a chromatin-binding-dependent mechanism in addition to *CDK4/6–Smad2/3* cross-talk

**Figure 2.**
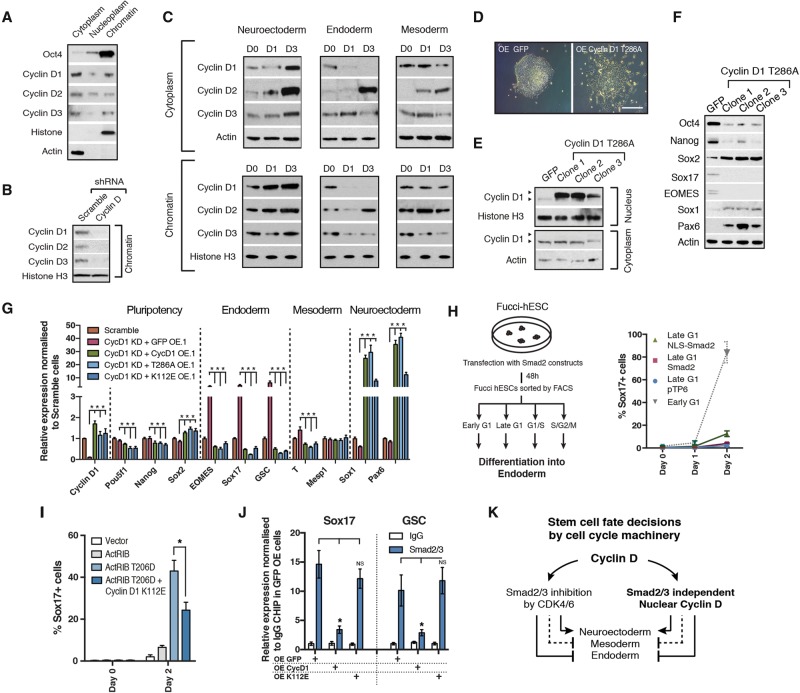
Nuclear Cyclin D binds to developmental loci and induces neuroectoderm while blocking endoderm differentiation in hESCs. (*A*) Cyclin D is localized both in the cytoplasm and on chromatin in hESCs. Cytoplasm, nucleoplasm, and chromatin were isolated from H9 cells and analyzed for Cyclin D1–3 protein by Western blot. Oct4 was used as a positive control. (*B*) Cyclin D knockdown in chromatin fractions. Chromatin fractions isolated from H9 cells expressing Scramble, Cyclin D1, Cyclin D2, or Cyclin D3 shRNA were analyzed for Cyclin Ds by Western blot. (*C*) Cyclin D cellular location during differentiation. H9 hESCs were differentiated into neuroectoderm, endoderm, and mesoderm. Cytoplasm and chromatin fractions were isolated and analyzed for relative Cyclin D1–3 levels by Western blot. Actin and Histone H3 acted as loading controls for cytoplasm and chromatin, respectively. (*D*) Morphology of hESCs overexpressing the Cyclin D1 T286A (CycD1-T286A) mutant. Representative colonies of hESCs overexpressing GFP and CycD1-T286A. (*E*) The CycD1-T286A mutant accumulates in the nucleus of hESCs. Western blot of Cyclin D1 in the cytoplasmic fraction and nucleoplasm. (*F*,*G*) CycD1-T286A overexpression causes neuroectoderm differentiation. Expression of neuroectoderm markers in CycD1-T286A-overexpressing cells shown by Western blot (*F*) and quantitative PCR (qPCR) (*G*). Significant differences compared with overexpressing GFP and calculated by two-way ANOVA are marked. (*H*) Nuclear Smad2 cannot bypass endoderm inhibition in late G1 cells. (*Left* panel) Schematic overview of the experiment. Tra-1–60-positive Fucci hESCs were sorted into early G1, late G1, and S/G2/M phase and differentiated into endoderm. (*Right* panel) Fucci hESCs transfected with Smad2 constructs 48 h before cell sorting were selected for 12 h with the goal of removing nontransfected cells and then sorted into late G1 phase and analyzed for marker expression by flow cytometry 1 or 2 d after endoderm differentiation. (*I*) CycD1-K112E cells can block endoderm and induce neuroectoderm differentiation in the presence of constitutively activated Activin–Smad2/3 signaling. Analysis of Sox17 expression by flow cytometry in cells transfected with ActRIB-expressing constructs. Significant differences calculated by *t*-test are marked. (*J*) CycD1-K112E regulates differentiation without affecting Smad2/3 binding to its target loci. Smad2/3 chromatin immunoprecipitation (ChIP) was performed in cells expressing GFP, Cyclin D1, or CycD1-K112E. Significant differences calculated by *t*-test are marked. (*K*) Schematics of dual mechanistically distinct functions for Cyclin D proteins in stem cell fate decisions. Cyclin Ds can repress endoderm in the cytoplasm by inhibiting Smad2/3 via CDK4/6 activation as shown before. Cyclin D proteins can also regulate cell fate decisions in stem cells in the nucleus that are independent of CDK4/6 and Smad2/3. All data are shown as mean ± SD. *n* = 3.

The above results suggest the existence of cell-autonomous mechanisms allowing Cyclin D1 to direct cell fate choice. Interestingly, studies in mouse retinal tissue and mouse cancer lines have shown that Cyclin D1 can participate in transcriptional regulation (Yu et al. 2005; Casimiro et al. 2012). However, whether this cell cycle regulator could also have a similar role in pluripotency exit and stem cell differentiation is unknown. Hence, we decided to explore whether similar mechanisms could occur in hESCs and could help to explain the CDK4/6-independent function of Cyclin D1 in neuroectoderm specification. For that, we performed Western blot analyses to determine the subcellular localization of Cyclin D proteins in hESCs and during their differentiation. These analyses revealed that Cyclin D1–3 not only localize to cytoplasm but also reside on chromatin in pluripotent cells (Fig. 2A,B). Cyclin D1–3 could also be found on the chromatin of neuroectodermal derivatives (Fig. 2C) and, to a lesser extent, in endoderm/mesoderm cells (Fig. 2C; Supplemental Fig. S2A,B). Together, these observations suggest that Cyclin Ds could indeed have a function on chromatin in hESCs.

To further uncover the mechanisms involving Cyclin Ds in the nucleus, we decided to perform a series of functional experiments focusing on Cyclin D1, since this is the most abundant Cyclin D on the chromatin while having the strongest effect on neuroectoderm differentiation (Pauklin and Vallier 2013; data not shown). We first overexpressed in hESCs a mutant form of Cyclin D1 (Cyclin D1 T286A [CycD1-T286A]) that accumulates in the nucleus due to the absence of phosphorylation of the residue T286 (Fig. 2D,E; Alt et al. 2000). CycD1-T286A hESCs showed a pronounced loss in pluripotency and mesoderm/endoderm gene expression and an increased neuroectoderm gene expression when compared with GFP or wild-type Cyclin D1 hESCs (Fig. 2F,G; Supplemental Figs. S2C–G, S3A–F). Furthermore, the effects of CycD1-T286A were maintained even with CDK4/6 inhibition (Supplemental Fig. S3G). Collectively, these results confirm that Cyclin D1 can influence cell fate decisions through nuclear mechanisms independently of CDK4/6.

Since Cyclin D1 controls accumulation of Smad2/3 into the cytoplasm during the late G1 phase of the cell cycle, we decided to determine whether the nuclear function of Cyclin D1 could be independent of the Activin/Nodal signaling pathway (Pauklin and Vallier 2013). FUCCI hESCs transitorily transfected with Flag-NLS-Smad2/3 (Lo and Massague 1999) could not overcome the inhibition of endoderm differentiation in late G1 (Fig. 2H), suggesting that forced nuclear import of Smad2/3 is not sufficient to bypass the mechanisms preventing this differentiation. Furthermore, inhibition of CDK4/6 results in only a limited increase in Sox17-expressing cells in endoderm differentiation induced in late G1, confirming the existence of Smad2/3 phosphorylation-independent mechanisms (Fig. 2H; Supplemental Fig. S3H–J). Finally, CycD1-K112E was able to significantly reduce the activity of a constitutive form of Activin receptor, ActRIB (ActRIB-T206A; *P* ≤ 0.05) (Fig. 2I), without affecting the binding of Smad2/3 to its target loci (Fig. 2J). Taken together, these data demonstrate that Cyclin D1 has a nuclear function in hESCs that promotes neuroectoderm and blocks endoderm specification independently of CDK4/6-mediated inhibition of Activin/Nodal–Smad2/3 signaling (Fig. 2K).

### Genome-wide identification of *Cyclin D1* target genes regulating stem cell differentiation

The functional studies described above support the hypothesis that Cyclin D1 functions via chromatin binding for controlling cell fate choice in hESCs. Previous studies on Flag-tagged Cyclin D1 in mice have shown that this protein can regulate gene expression by interacting with transcription factors and epigenetic modifiers in cancer cells and mouse embryos (Fu et al. 2005a,b; Landis et al. 2006; Geng et al. 2007; Sicinski et al. 2007; Bienvenu et al. 2010; Casimiro et al. 2012). Consequently, we hypothesized that Cyclin D1's role in the nucleus could be to control transcription of genes that are involved in cell fate decisions of hESCs.

**Figure 3.**
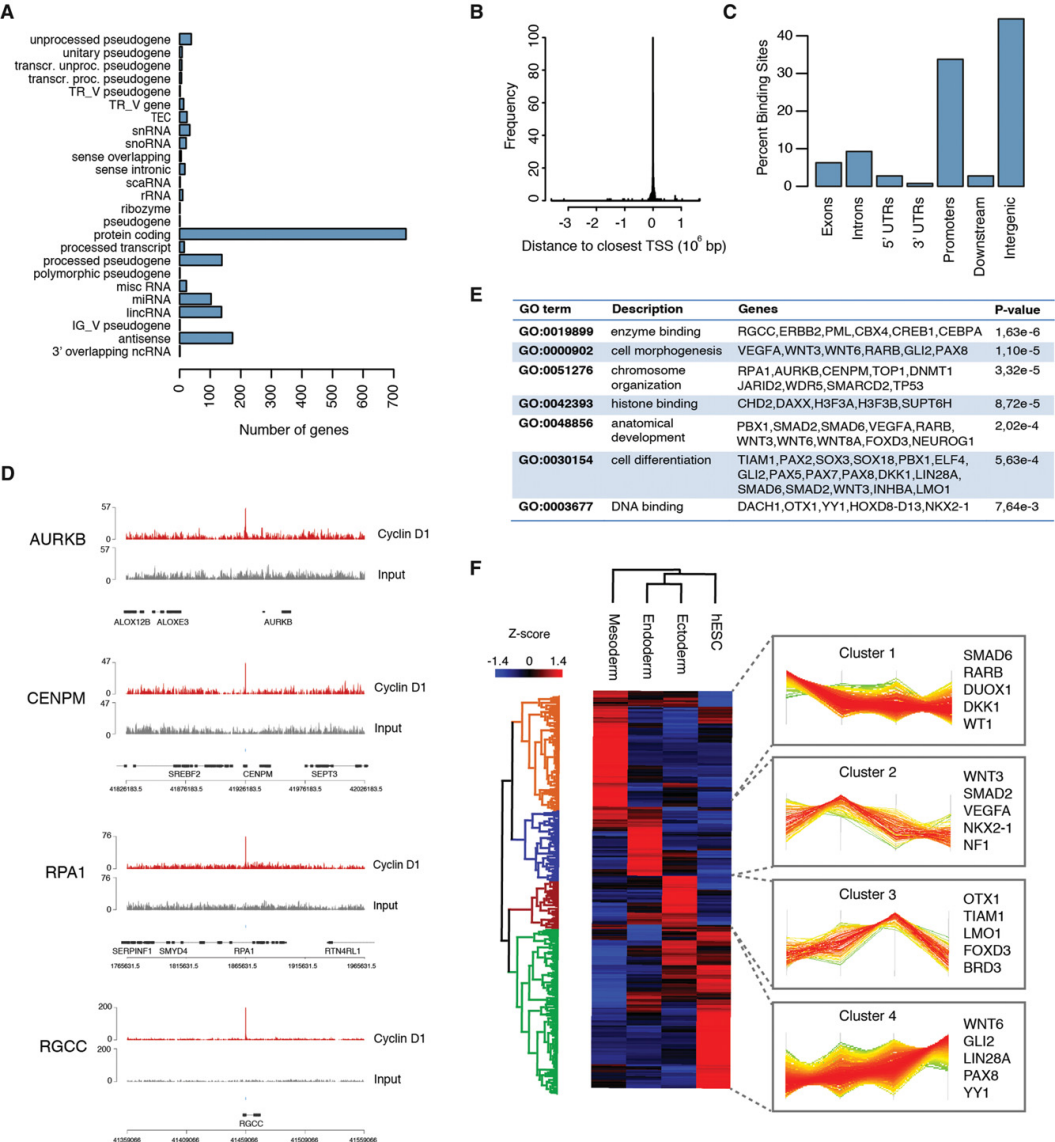
Genome-wide detection of DNA-binding occupancy for endogenous Cyclin D1 in hESCs. (*A*) The function of genes spanning 50 kb upstream of and 50 kb downstream from Cyclin D1-binding peaks. Classification of the genes close to Cyclin D1-binding regions identified by ChIP-seq. (*B*) The localization of Cyclin D-binding peaks. The distance of Cyclin D1 peaks relative to the transcription start site (TSS) of surrounding genes. (*C*) Peak annotation of Cyclin D-binding regions on its target genes. Promoter regions span 10 kb upstream of the transcription start site, while immediate downstream regions span 5 kb after 3′ untranslated regions (UTRs). (*D*) Cyclin D1-binding site close to the cell cycle regulator genes RGCC and RPA1 and chromosome segregation factors AURKB and CENPM, which are also Cyclin D1 targets in mice. Genomic region 50 kb upstream of and 50 kb downstream from the binding peak. (*E*) Cyclin D1 targets include developmental loci and genes involved in differentiation. Selected gene ontology terms of genes surrounding Cyclin D1-binding regions. (*F*) Euclidian *k*-means (*k* = 4) hierarchical clustering of RNA sequencing (RNA-seq) data in the undifferentiated, neuroectoderm, mesoderm, and endoderm germ layer for Cyclin D1-bound protein-coding genes identified by ChIP-seq. Cyclin D1 protein-coding target genes (740) were associated (677 of 740) with FPKM (fragments per kilobase per million mapped fragments) values and differential expression analysis of RNA-seq (HUES64 vs. endoderm, HUES64 vs. mesoderm, or HUES64 vs. ectoderm) for all protein-coding genes expressed in hESCs and the differentiated populations (Supplemental Table S2; Gifford et al. 2013). *Z*-scores indicate the differential expression measured in the number of standard deviations from the average level across all germ layer and hESC conditions. Representative developmental genes with a significant change in expression are listed for each gene cluster.

To investigate this potential function, we performed genome-wide identification of endogenous Cyclin D1-binding sites in hESCs using chromatin immunoprecipitation (ChIP) followed by high-throughput sequencing (ChIP-seq). We identified 400 significantly read-enriched genomic regions (or peaks) to be reproducible between two independent biological replicates (*P* ≤ 1 × 10^−5^; false discovery rate [FDR] ≤0.01). Peaks were associated with 1519 target genes within regions spanning 50 kb upstream of and 50 kb downstream from the Cyclin D1 peaks, and about half of the genes are protein-coding (Fig. 3A; Supplemental Table S1). The majority of the peaks were overlapping with or located close to transcription start sites (Fig. 3B; Supplemental Tables S2, S3), and >30% of the Cyclin D1-binding sites in hESCs were located in proximal promoter regions within 5 kb of genes (Fig. 3C). Interestingly, Cyclin D1 also binds a large number of intergenic regions >10 kb upstream of or downstream from genes, which could represent enhancers and regulatory regions, since a proportion of these overlap with H3K4me1 and H3K27ac regions. Thus, Cyclin D1 could also control gene expression via long-distance interactions of enhancer–promoter regions. Together, these observations reinforce our hypothesis concerning the involvement of Cyclin D1 in transcriptional regulation of genes in hESCs.

To investigate the putative functional pathways of the Cyclin D1 target genes, we performed gene ontology (GO) analysis. GO terms associated with the 740 protein-coding genes were significantly enriched for terms related to enzyme binding (*P* = 1.63 × 10^−6^) and chromosome organization (*P* = 3.32 × 10^−5^) (Supplemental Table S4). Representative Cyclin D1 target genes were chromosome segregation factors such as *AURKB* and *CENPM* (Fig. 3D) that have previously been identified as targets of Cyclin D1 in mouse embryonic fibroblasts (Casimiro et al. 2012) and have been implicated in chromosome instability in cancers (Thompson et al. 2010; Casimiro and Pestell 2012; Pestell 2013). Among the target genes were also replication protein *RPA1,* cell cycle regulator *RGCC* (Fig. 3D; Supplemental Table S4), and DNA topoisomerase *TOP1*. Importantly, the GO terms enriched for Cyclin D1 target loci also included cell morphogenesis (*P* = 1.1 × 10^−5^), anatomical development (*P* = 2.2 × 10^−4^), cell differentiation (*P* = 5.63 × 10^−4^), and DNA binding (*P* = 7.64 × 10^−3^) (Fig. 3E). These results suggest that Cyclin D1 could act as a transcriptional regulator during cell fate choice of stem cells.

### *Cyclin D1* binds to its developmental target loci in a cell cycle-dependent manner

To further determine whether Cyclin D1 target genes included regulators of cell fate decisions, we compared their expression levels in endoderm, mesoderm, and neuroectoderm using published RNA sequencing (RNA-seq) data (Fig. 3F; Gifford et al. 2013). We did not observe an overall uniform change in expression of all Cyclin D1 target genes, suggesting that Cyclin D1 does not act as a genome-wide repressor or activator of transcription (Supplemental Fig. S4B). However, Cyclin D1 could act as a locus-specific regulator of developmental genes, as, among its target loci, we found important inducers of germ layer specification that showed up-regulation primarily in neuroectoderm (cluster 3: *OTX1*, *LMO1*, and *TIAM1*) (Fig. 3F; Supplemental Table S5), endoderm (cluster 2: *Wnt3*, *Smad2*, and *VEGFA*) (Fig. 3F), or mesoderm (cluster 1: *RARB*, *DKK1*, and *Smad6*) (Fig. 3F) as well as genes that indicate a down-regulation upon stem cell differentiation to all three germ layers (cluster 4: *Wnt6*, *GLI2*, and *LIN28A*) (Fig. 3F).

**Figure 4.**
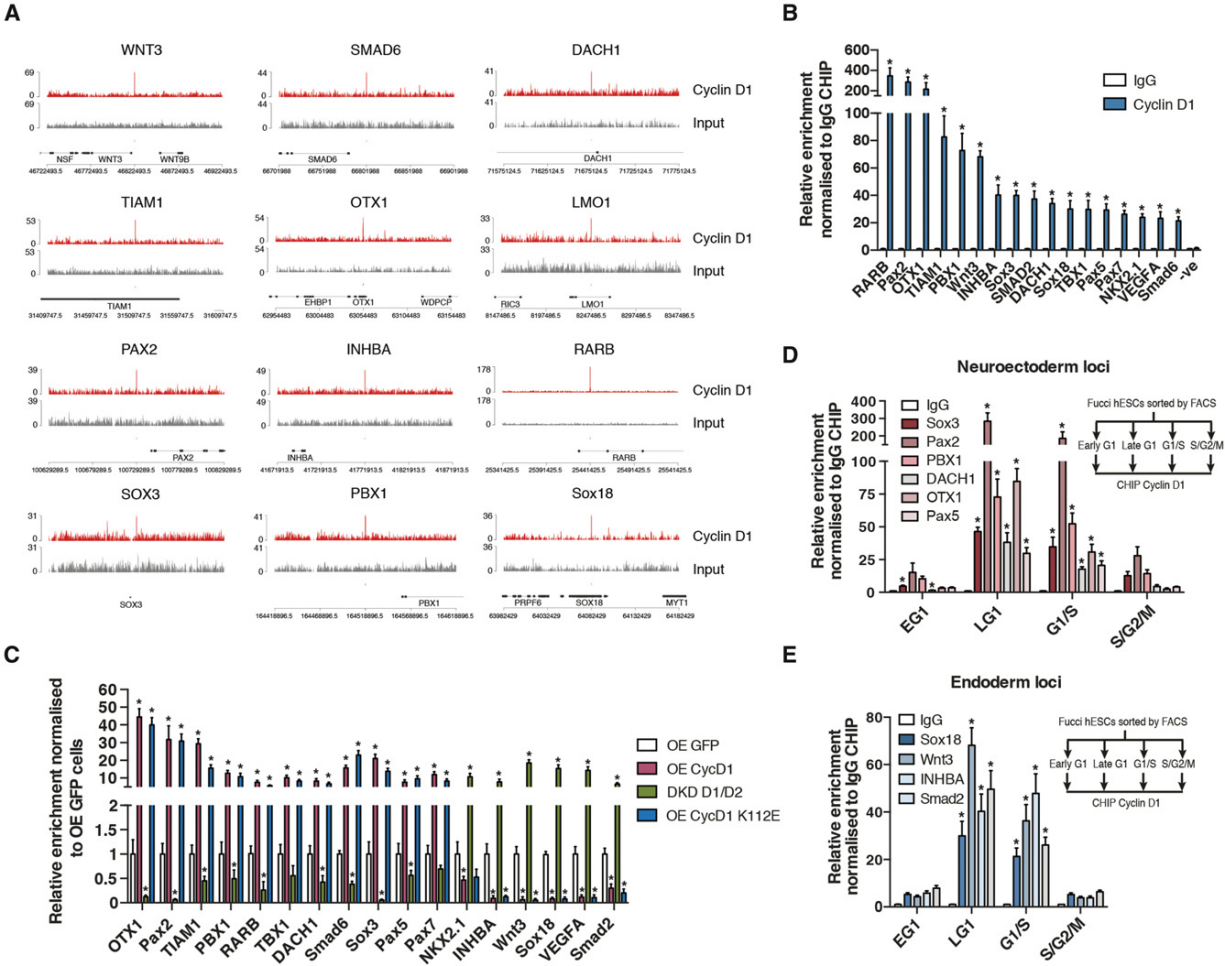
Cyclin D1 binds to its developmental target genes in a cell cycle-dependent manner. (*A*) Representative Cyclin D1-binding regions close to developmental genes spanning 50 kb upstream of and 50 kb downstream from each ChIP-seq peak. Cyclin D1 binding was identified by ChIP-seq analysis of endogenous Cyclin D1 in hESCs. (*B*) Cyclin D1 ChIP-qPCR at genomic regions close to selected developmental loci that were identified as Cyclin D1-binding sites by ChIP-seq. Significant differences calculated by *t*-test are marked. (*C*) Cyclin D1 overexpression induces its neuroectodermal target genes while reducing its endoderm target genes. Gene expression changes of Cyclin D target genes in Cyclin D1-overexpressing cells, CycD1-K112E-overexpressing cells, and Cyclin D1/Cyclin D2 double-knockdown cells expressing shRNAs compared with control cells expressing GFP. Significant differences compared with GFP overexpression and calculated by *t*-test are marked. (*D*,*E*) Cell cycle-dependent binding of Cyclin D1 to neuroectoderm genes (*D*) and endoderm genes (*E*). Cyclin D1 ChIP was performed on Tra-1–60-positive FACS-sorted Fucci hESCs, and then qPCR was performed to detect the genes denoted. Significant differences compared with G2/M phase and calculated by *t*-test are marked. All data are shown as mean ± SD. *n* = 3.

Since Cyclin D1 target genes included regulators of germ layer specification such as transcription factors and components of signaling pathways (*Wnt*, *Bmp4*, and *Activin/Nodal/TGFβ*) (Pauklin and Vallier 2015), we decided to focus on a subset of these genes that showed a strong enrichment in Cyclin D1 binding in ChIP-seq analysis (Fig. 4A; Supplemental Fig. S4A–C). First, we confirmed the binding of Cyclin D1 to these genomic regions (Fig. 4B; Supplemental Fig. S4D). Of note, we also showed the absence CDK6 on the same genome regions by ChIP and quantitative PCR (ChIP-qPCR) (Supplemental Fig. S5A), thereby confirming that Cyclin D control of transcription is independent of its phosphorylation activity. Furthermore, these developmental target genes showed distinct expression patterns in hESCs differentiated into three germ layers using defined culture condition (Supplemental Fig. S5B,C). Importantly, the endoderm-specific genes were repressed by Cyclin D1 overexpression, whereas the neuroectoderm-specific genes were induced (Fig. 4C). Furthermore, Cyclin D1/Cyclin D2 double knockdown resulted in opposite changes of expression for these genes when compared wth overexpression (Fig. 4C), suggesting that Cyclin D1 could control stem cell fate choice by transcriptional induction of neuroectoderm genes (*Pax2*, *Sox3*, *PBX1*, *DACH1*, and *Otx1*) and transcriptional repression of endoderm genes (*Sox18*, *Wnt3*, and *Smad2*). Finally, we tested the binding of Cyclin D1 to its target loci in FUCCI hESCs sorted to distinct cell cycle phases. Since Cyclin Ds are specifically expressed in late G1, we hypothesized that Cyclin D1 would bind those genomic regions in specific phases of the cell cycle. Accordingly, ChIP-qPCR analyses demonstrated that Cyclin D1 binds promoters of neuroectoderm (Fig. 4D) and endoderm (Fig. 4E) factors specifically during the late G1 phase and G1/S transition. These data collectively suggested that Cyclin D1 could control the transcriptional activity of developmental genes by binding proximal regulatory regions in a cell cycle-dependent manner.

### *Cyclin D1* recruits transcriptional coregulators to control the expression of developmental genes in stem cells

**Figure 5.**
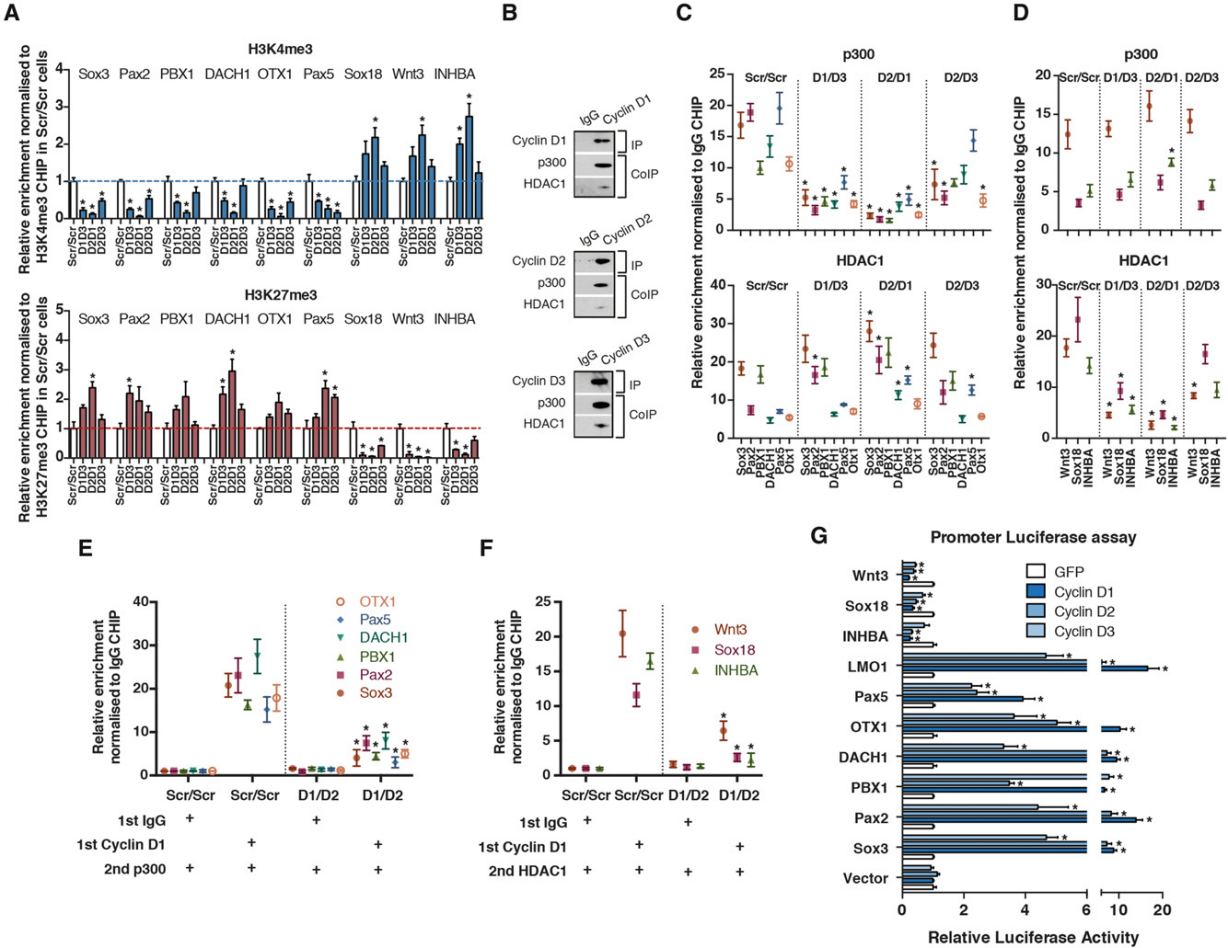
Cyclin Ds interact with transcriptional coregulators to control germ layer specification genes. (*A*) Cyclin D expression promotes the deposition of active histone mark H3K4me3 on neuroectoderm genes and repressive histone mark H3K27me3 on endoderm genes. ChIP of histone H3K4me3 and H3K27me3 was performed on Tra-1–60-positive Cyclin D double-knockdown hESCs, and then qPCR was performed to detect genomic regions corresponding to selected key developmental genes. hESCs expressing Scramble shRNA (Scr/Scr) were used as a control. The dashed line indicates no change with respect to Scr/Scr ChIP. Significant differences compared with Scr/Scr and calculated by *t*-test are marked. (*B*) Cyclin D1–D3 interact with p300 and HDAC1 on chromatin of hESCs. Cyclin Ds were immunoprecipitated from chromatin fractions and analyzed for the presence of p300 and HDAC1 by Western blot. (*C*,*D*) Cyclin D proteins recruit transcriptional coregulators to loci controlling germ layer specification. ChIP of coactivator p300 and corepressor HDAC1 was performed on Tra-1–60-positive Cyclin D double-knockdown hESCs, and then qPCR was performed to detect genomic regions corresponding to developmental genes regulating neuroectoderm (*C*) and endoderm (*D*) specification. hESCs expressing Scramble shRNA (Scr/Scr) were used as a control. Significant differences compared with Scr/Scr and calculated by *t*-test are marked. (*E*,*F*) Cyclin D is necessary to recruit p300 to neuroectoderm loci and HDAC1 to endoderm loci in hESCs. Sequential ChIP was carried out in Scr/Scr and Cyclin D1/D3 double-knockdown cells by first performing a Cyclin D1 ChIP followed by p300 or HDAC1 ChIP on neuroectoderm (*E*) and endoderm (*F*) loci. Significant differences compared with Scr/Scr and calculated by *t*-test are marked. (*G*) Cyclin Ds can induce transcriptional activity of neuroectoderm genes and repress transcriptional activity of endoderm genes. A luciferase expression construct containing promoter regions of the developmental genes with Cyclin D1-binding sites was transfected together with Cyclin D1–3 and analyzed for luciferase activity 48 h after transfection. Renilla luciferase was used as an internal control during transfections. Significant differences compared with GFP overexpression and calculated by *t*-test are marked. All data are shown as mean ± SD. *n* = 3.

To understand the mechanisms involved in Cyclin D1-dependent transcriptional regulation, we analyzed the impact of Cyclin D1 on the epigenetic landscape of its target genes. The knockdown of Cyclin D1 resulted in the loss of the histone modification H3K4me3 on neuroectoderm loci but not on endoderm loci (Fig. 5A). Thus, Cyclin D1 could be necessary for the deposition of this positive epigenetic mark. Interestingly, Cyclin D1 has previously been shown to interact with transcriptional coactivator and histone modifier p300 on the PPARγ-responsive element of the lipoprotein lipase promoter during adipocyte formation (Kim et al. 2000; Fu et al. 2005a,b). We therefore performed coimmunoprecipitations (co-IPs) between Cyclin Ds and p300 from the chromatin fraction of hESCs, which indicated that these proteins can also form a complex in stem cells (Fig. 5B). Protein truncation analyses further showed that these interactions involved the domain of Cyclin D1 spanning residues 91–179, thereby confirming a direct interaction (Supplemental Fig. S6A). Finally, ChIP analyses showed that p300 recruitment to neuroectodermal loci was impaired in hESCs knocked down for the expression of Cyclin Ds, whereas overexpression of Cyclin D1 increases the presence of p300 on these loci (Fig. 5C; Supplemental Fig. S6B–D).

In contrast to H3K4me3, the knockdown of Cyclin Ds led to the loss of the repressive histone mark H3K27me3 in endoderm loci (Fig. 5A). In agreement with this and with a previous study (Fu et al. 2005a), we also found that Cyclin Ds can interact with the transcriptional repressor HDAC1 (Fig. 5B), and Cyclin D knockdown causes its loss on endoderm loci (Fig. 5D). We performed sequential ChIP of Cyclin D1 and HDAC1 or p300, validating the simultaneous binding of these transcriptional and cell cycle regulators on developmental gene promoters (Fig. 5E,F), while Cyclin D1 overexpression in hESCs increases HDAC1 binding to endoderm loci (Supplemental Fig. S6E,F). Altogether, these results imply that Cyclin Ds bind the promoters of developmental genes in stem cells, influencing their epigenetic status by interacting with epigenetic modifiers.

Finally, we validated the functional importance of these interplays by generating a luciferase reporter system with regulatory regions bound by Cyclin D1 on its corresponding target loci. We cotransfected these luciferase constructs into hESCs with Cyclin D1–3 and analyzed promoter activities after 48 h. These experiments showed that Cyclin Ds could activate reporters for neuroectoderm genes (Fig. 5G), indicating the capacity of these cell cycle regulators to induce transcription. On the other hand, we observed that overexpression of Cyclin Ds limits their transcriptional activity of endoderm genes, confirming that these cell cycle regulators could also act as transcriptional repressors (Fig. 5G).

These data collectively demonstrate that Cyclin Ds control the expression of regulators of neuroectoderm and endoderm differentiation in hESCs by recruiting transcriptional coactivators and corepressors onto their promoters regions.

### *Cyclin D1* binds transcription factors to control the induction of endoderm and neuroectoderm genes in stem cells

**Figure 6.**
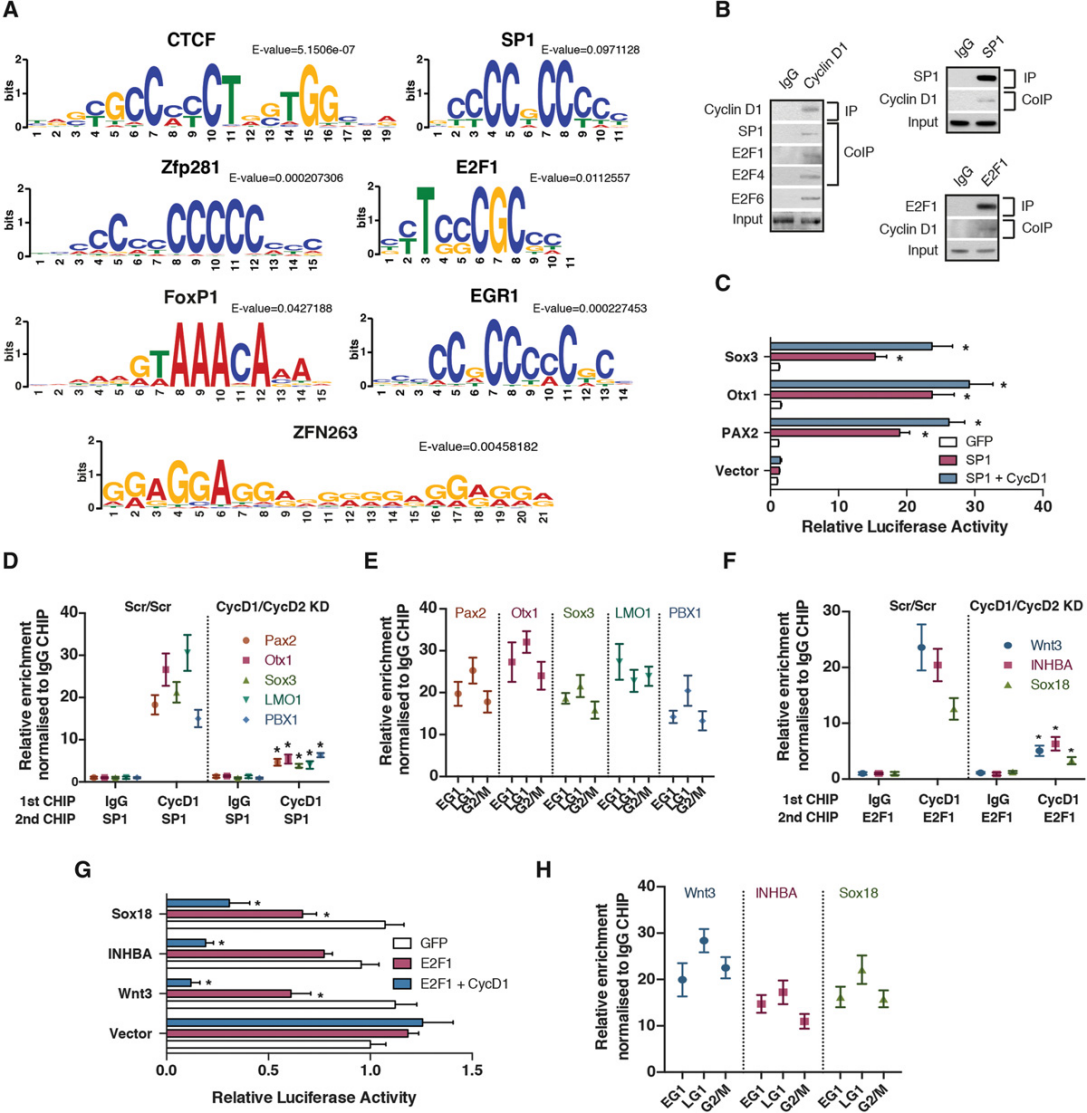
Cyclin D regulates transcriptional initiation by forming a complex with SP1 and E2F1 on developmental loci. (*A*) Comparison of consensus DNA sequence motifs identified in Cyclin D1-binding sites by MEME-ChIP with known DNA motifs using TOMTOM (Gupta et al. 2007). (*B*) Cyclin D binds to E2F1/4/6 and SP1 on chromatin of hESCs. Chromatin fractions were isolated from hESCs and used for immunoprecipitation studies. Coimmunoprecipitated proteins were detected by Western blotting. (*C*) A luciferase expression construct containing promoter regions of neuroectoderm genes with Cyclin D1-binding sites was cotransfected with SP1 or Cyclin D1 and analyzed for luciferase activity 48 h after transfection. Renilla luciferase was used as an internal control during transfections. Significant differences compared with GFP overexpression and calculated by *t*-test are marked. (*D*) Cyclin D forms a complex with SP1 on neuroectoderm loci. Sequential ChIP of Cyclin D1 and SP1 in Scrambled (Scr/Scr) and Cyclin D1/D2 double-knockdown cells. Significant differences compared with Cyclin D1 ChIP/SP1 ChIP in Scrambled (Scr/Scr) cells and calculated by *t*-test are marked. (*E*) SP1 binds to neuroectoderm promoters throughout the cell cycle. Fucci hESCs were sorted into different cell cycle phases and analyzed by SP1 ChIP. (*F*) Cyclin D1 forms a complex with E2F1 on endoderm loci Wnt3, INHBA, and Sox18. Sequential ChIP of Cyclin D1 and E2F1 in Scrambled (Scr/Scr) and Cyclin D1/D2 double-knockdown cells. Significant differences compared with Cyclin D1 ChIP/E2F1 ChIP in Scrambled (Scr/Scr) cells and calculated by *t*-test are marked. (*G*) A luciferase expression construct containing regulatory regions of endoderm genes with Cyclin D1-binding sites was transfected together with E2F1 or cotransfected with Cyclin D1 and analyzed for luciferase activity 48 h after transfection. Renilla luciferase was used as an internal control during transfections. Significant differences compared with GFP overexpression and calculated by *t*-test are marked. (*H*) E2F1 binds to endoderm promoters Wnt3, INHBA, and Sox18 throughout the cell cycle. Fucci hESCs were sorted into different cell cycle phases and analyzed by E2F1 ChIP. All data are shown as mean ± SD. *n* = 3.

Cyclin D1 is likely to recruit epigenetic regulators onto distinct genomic regions by cooperating with sequence-specific transcription factors. To identify its potential coregulators, we performed de novo motif discovery analysis followed by a comparison of detected motifs against databases using MEME-ChIP (Machanick and Bailey 2011) on the DNA sequences associated with Cyclin D1-binding sites. The most significantly overrepresented motifs found corresponded to SP1, E2Fs, EGR1, Zfp281, FoxP1, CTCF, and Zfn263 (Fig. 6A; Supplemental Fig. S7A; full list in Supplemental Table S7). Next, we decided to characterize the functional cooperation between Cyclin D1 and the identified transcription factors in more detail, focusing on SP1 and E2F1/4/6 as being among the top hits identified by the transcription factor motif comparison. Co-IP experiments verified that Cyclin D1 and SP1 could be found in the same protein complexes on chromatin in hESCs (Fig. 6B). Transient overexpression of SP1 in hESCs resulted in the induction of *SOX3*, *OTX1*, and *PAX2* reporter genes, suggesting that the binding of SP1 on these neuroectodermal gene promoters could be functional. Of note, sequential ChIP with Cyclin D1 and SP1 confirmed the interactions between these two proteins on DNA (Fig. 6D). Finally, Cyclin D1 overexpression did not alter the presence of SP1 (Supplemental Fig. S7B) on neuroectodermal promoters, while SP1 binding to neuroectoderm loci did not change significantly during the cell cycle (Fig. 6E).

Concerning endoderm genes, co-IP (Fig. 6B; Supplemental Fig. S7D,E) and sequential ChIP (Fig. 6F) experiments confirmed that Cyclin D1 and E2Fs could be found in the same protein complex on *Wnt3*, *Sox18*, and *INHBA* regulatory regions. Transient overexpression of E2F1 was able to reduce the activity of endoderm reporter genes, while cotransfection of Cyclin D1 further strengthened this repressive effect (Fig. 6G), thereby implying that E2F1 and Cyclin D1 binding could be functional. The increase or decrease in Cyclin D1 expression did not affect the presence of E2F1 on *Wnt3*, *Sox18*, and *INHBA* promoters (Supplemental Fig. S7C), and E2F1 binding to DNA was not cell cycle-dependent (Fig. 6H). Therefore, E2F1 could bind endodermal promoters independently of cell cycle regulations. These results suggest that E2F1 is involved in transcriptionally repressive interaction with Cyclin D1 protein specifically in late G1.

**Figure 7.**
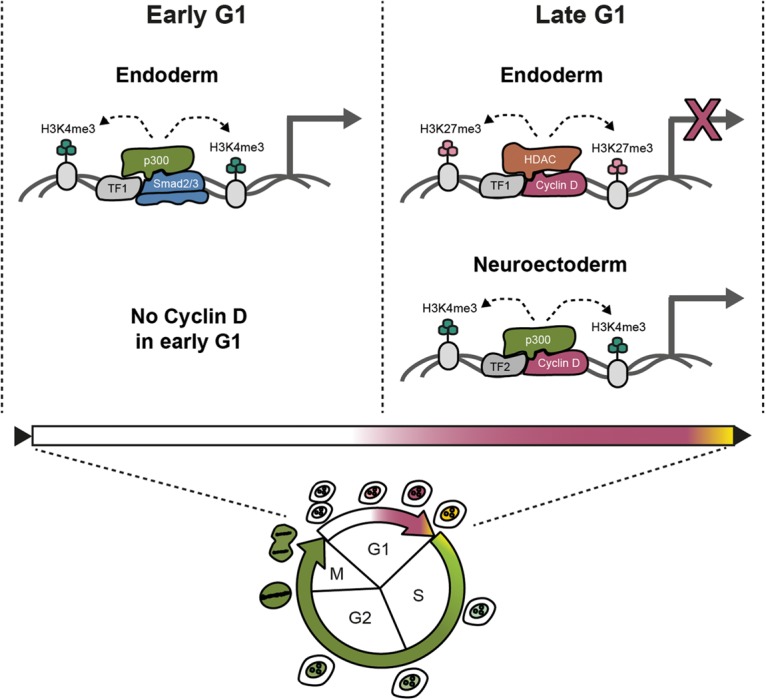
Transcriptional regulation of developmental genes by Cyclin D proteins during cell cycle progression of human pluripotent stem cells. Cyclin D binds to SP1 and recruits p300 to neuroectoderm loci for promoting their transcriptional initiation. In contrast, Cyclin D binds to E2Fs and recruits HDAC1 to endoderm loci for blocking their transcriptional initiation.

Taken together, these results reveal that the activity of transcription factors can be cell cycle-regulated through their interactions with Cyclin D1 (Fig. 7) and that this regulation establishes the epigenetic modifications necessary for transcriptional initiation of developmental master regulators during primary germ layer specification.

## Discussion

Here we uncovered that, in addition to their cross-talk with the signaling pathway Activin/Nodal–Smad2/3, the mitogenic sensor Cyclin D1 also orchestrates cell fate decisions of stem cells via a complementary mechanism that involves a direct transcriptional regulation of developmental master regulators (Fig. 7). These results reveal the mechanisms by which cell fate decisions are initiated during the narrow opportunity window of G1 phase when Cyclin Ds are dynamically expressed. Accordingly, endoderm induction occurs in early G1 when the level of Cyclin Ds is low, allowing Smad2/3 to bind and activate endoderm genes. In late G1, Cyclin D1 is induced and forms a complex with locus-specific transcription factors to recruit transcriptional coactivators onto neuroectoderm genes and corepressors onto endoderm genes. This model explains the molecular mechanisms allowing cell cycle regulators to directly control differentiation by regulating transcription factor activity during cell cycle progression and provides further support for cell cycle-coordinated cell fate decisions in stem cells.

Importantly, similar cell cycle-dependent transcriptional mechanisms converging on developmental factors could apply to other cell cycle regulators in pluripotent cells. For instance, RB genes (pRB, Rbl1, and Rbl2) affect differentiation in mouse ESCs (mESCs) and hESCs (Conklin et al. 2012). Furthermore, the CDK inhibitor p27 cooperates with Rbl2 to repress Sox2 expression during differentiation of mESCs (Li et al. 2012) while promoting the expression of mesoderm markers *brachyury* and *twist* in hESCs (Menchon et al. 2011). Transcriptional regulation of developmental genes by cell cycle regulators could therefore be a common mechanism, and a complex cross-talk is likely to occur between these proteins.

Our results also uncover the mechanisms by which cell cycle regulators are recruited to specific genomic locations in hESCs. Indeed, we performed for the first time a genome-wide survey of Cyclin D1 location on the stem cell chromatin, and this analysis proved that Cyclin Ds have the capacity to interact with transcription factors SP1 and E2F1. Interestingly, SP1 knockout mice have reduced embryo size and a diversity of phenotypes that are lethal before 9.5 d post-coitum (dpc) (Marin et al. 1997). These observations suggest that SP1 is involved in controlling cell proliferation and tissue differentiation in the early embryo and thus that the mechanisms described in our model could be relevant for normal development. The importance of E2F1 in vivo is more complicated to establish, since E2F genes are known to be functionally redundant during embryonic development (Tsai et al. 2008). However, pRB and E2F family members can control neuronal and pancreatic development by direct regulation of genes such as Ngn3 (Kim and Rane 2011; Tsume et al. 2012). Thus, E2F factors could orchestrate stem cell and progenitor differentiation in a diversity of tissues, underlining that cell cycle control of cell fate choice could be a common mechanism between a diversity of progenitors.

The absence of Cyclin Ds or CDK4/6 has only modest effects on early mouse development despite their universal function in primary and cancer cells. Functional redundancy between cell cycle regulators is frequently mentioned to explain this relative lack of role in vivo (Jena et al. 2002; Kozar et al. 2004; Yu and Sicinski 2004; Ciemerych and Sicinski 2005). Nevertheless, Cyclin Ds have a tissue-specific expression during gastrulation (Wianny et al. 1998) that resembles the pattern observed during differentiation of hESCs in vitro, suggesting that their role in the mechanisms controlling early cell fate decisions could be conserved in vivo. Cyclin D activity also promotes neuroectoderm formation during induced pluripotent stem cell generation through up-regulating Pax6 (Chen et al. 2014), and, importantly, Cyclin D maintains the self-renewal of a broad number of adult stem cells, including mammary stem and progenitor cells (Jeselsohn et al. 2010), neuronal stem cells (Roccio et al. 2013), and hematopoietic stem cells (Lange and Calegari 2010). Therefore, the basic mechanisms uncovered by these studies could also be relevant for adult stem cells and represent an important step toward understanding the balance between differentiation and self-renewal during organ development and repair.

## Materials and methods

### Cell culture of hESCs

hESCs (H9 from WiCell) and mEpiSCs were grown in defined culture conditions as described previously (Brons et al. 2007). H9 cells were passaged weekly using collagenase IV and maintained in chemically defined medium (CDM) supplemented with 10 ng/mL Activin A and 12 ng/mL FGF2.

### Differentiation of hESCs

hESCs were differentiated into neuroectoderm, endoderm, and mesoderm as described previously (Vallier et al. 2009). Briefly, cells were cultured in CDM supplemented with 10 µM SB-431542 (Tocris) and 12 ng/mL FGF2 for neuroectoderm; in CDM + PVA supplemented with 100 ng/mL Activin A, 20 ng/mL FGF2, 10 ng/mL BMP4, 10 µM Ly294002 (Promega), and 3 µM CHIR99021 (Selleck) for mesoderm; and in CDM − PVA supplemented with 100 ng/mL Activin A, 20 ng/mL FGF2, 10 ng/mL BMP4, and 10 µM Ly294002 (Promega) for endoderm. hESCs were differentiated as described before (Pauklin and Vallier 2013).

### ChIP-seq

ChIP samples were sequenced at the Cambridge Institute Next-Generation Sequencing service of the University of Cambridge using Illumina HiSeq, and the raw data have been deposited on ArrayExpress under accession number E-MTAB-3807. Peak calling analysis (Bailey et al. 2013) was performed using PeakRanger (Feng et al. 2011), and only the peaks that were reproducible at an FDR of ≤0.01 in two biological replicates were selected for further processing.

Extended Materials and Methods with the associated references are in the Supplemental Material.

## Supplementary Material

Supplemental Material
