# Supplementary material for: Initiation of stem cell differentiation involves cell cycle-dependent regulation of developmental genes by Cyclin D
Source: Genes Dev. 2016 Feb 15;30(4):421–33. doi: 10.1101/gad.271452.115 (PMC4762427; doi:10.1101/gad.271452.115)
Supplement: Supplemental Material [file supp_30_4_421__index.html]

Supplemental Material 

# Initiation of stem cell differentiation involves cell cycle-dependent regulation of developmental genes by Cyclin D

## Supplemental Material

**Files in this Data Supplement:**

- Supp Fig S1.tif
- Supp Fig S2.tif
- Supp Fig S3.tif
- Supp Fig S4.tif
- Supp Table S7.xlsx
- Supp Fig S5.tif
- Supp Fig S6.tif
- Supp Fig S7.tif
- Supp Material.docx
- Supp Table S1.xlsx
- Supp Table S2.xlsx
- Supp Table S3.xlsx
- Supp Table S4.xlsx
- Supp Table S5.xlsx
- Supp Table S6.xlsx
