# Supplementary figures and images for: Initiation of stem cell differentiation involves cell cycle-dependent regulation of developmental genes by Cyclin D
Source: Genes Dev. 2016 Feb 15;30(4):421–33. doi: 10.1101/gad.271452.115 (PMC4762427; doi:10.1101/gad.271452.115)

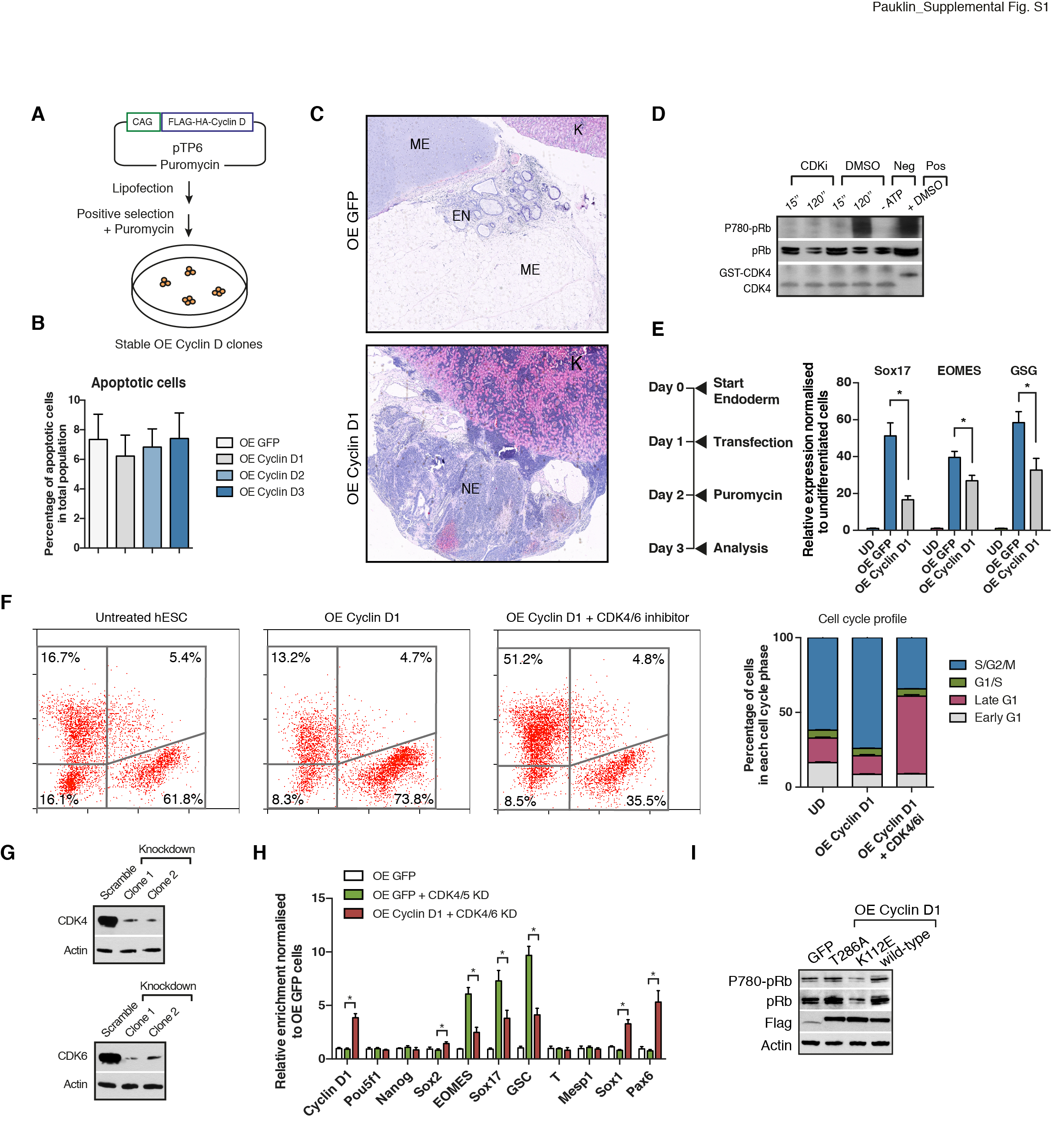

Supplement: Supplemental Material [file supp_30.4.421_Fig_S1.tif]

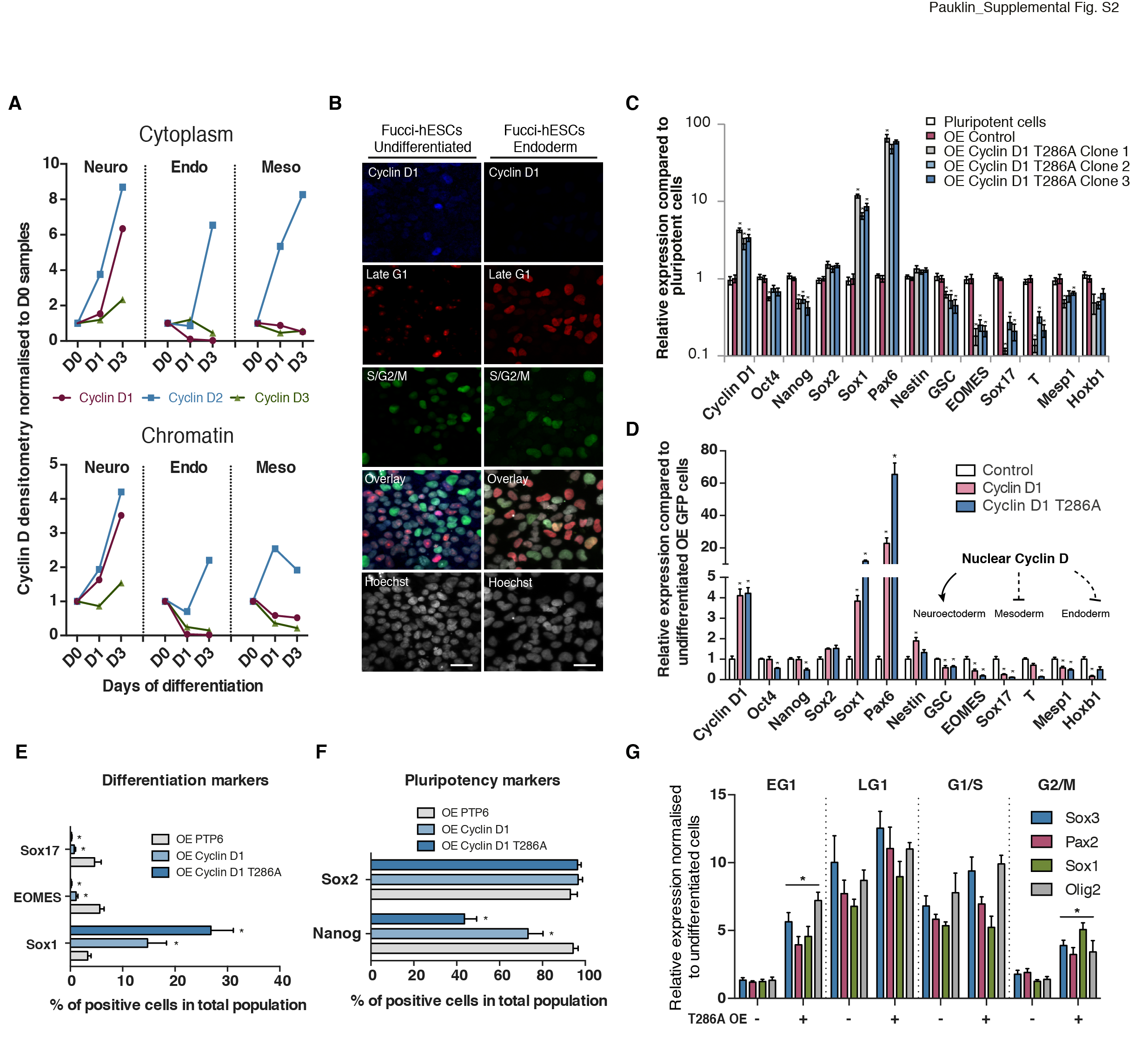

Supplement: Supplemental Material [file supp_30.4.421_Fig_S2.tif]

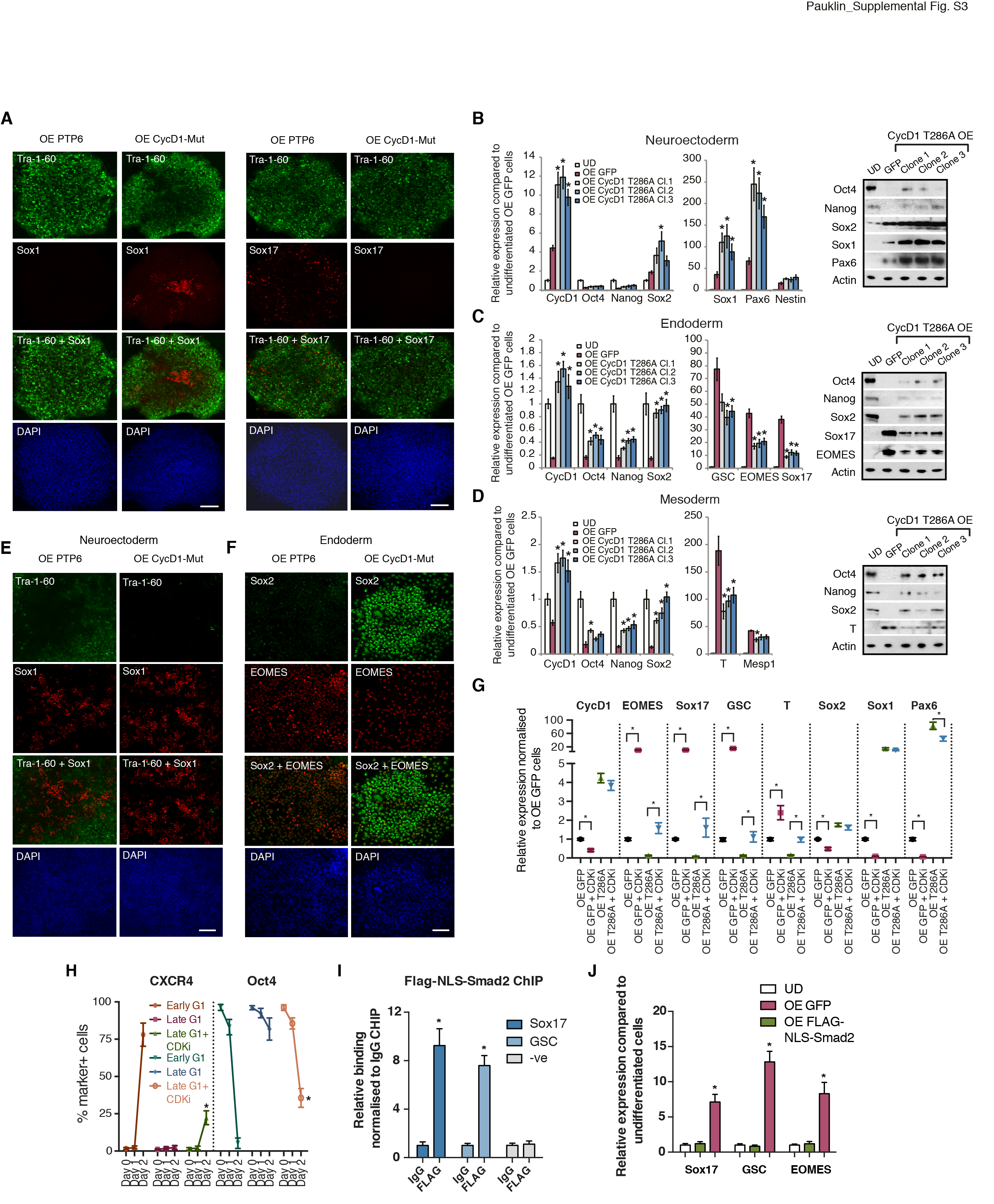

Supplement: Supplemental Material [file supp_30.4.421_Fig_S3.tif]

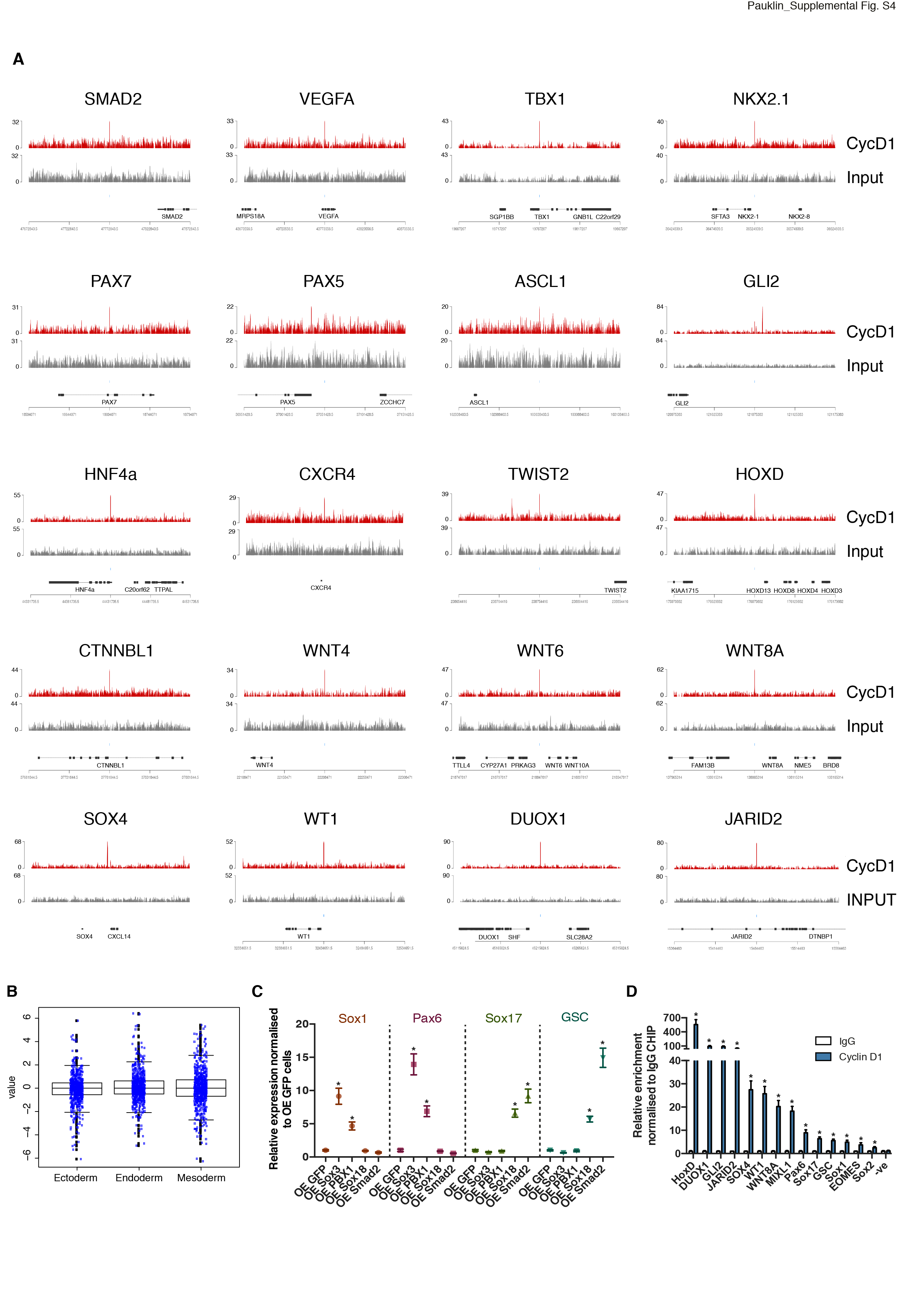

Supplement: Supplemental Material [file supp_30.4.421_Fig_S4.tif]

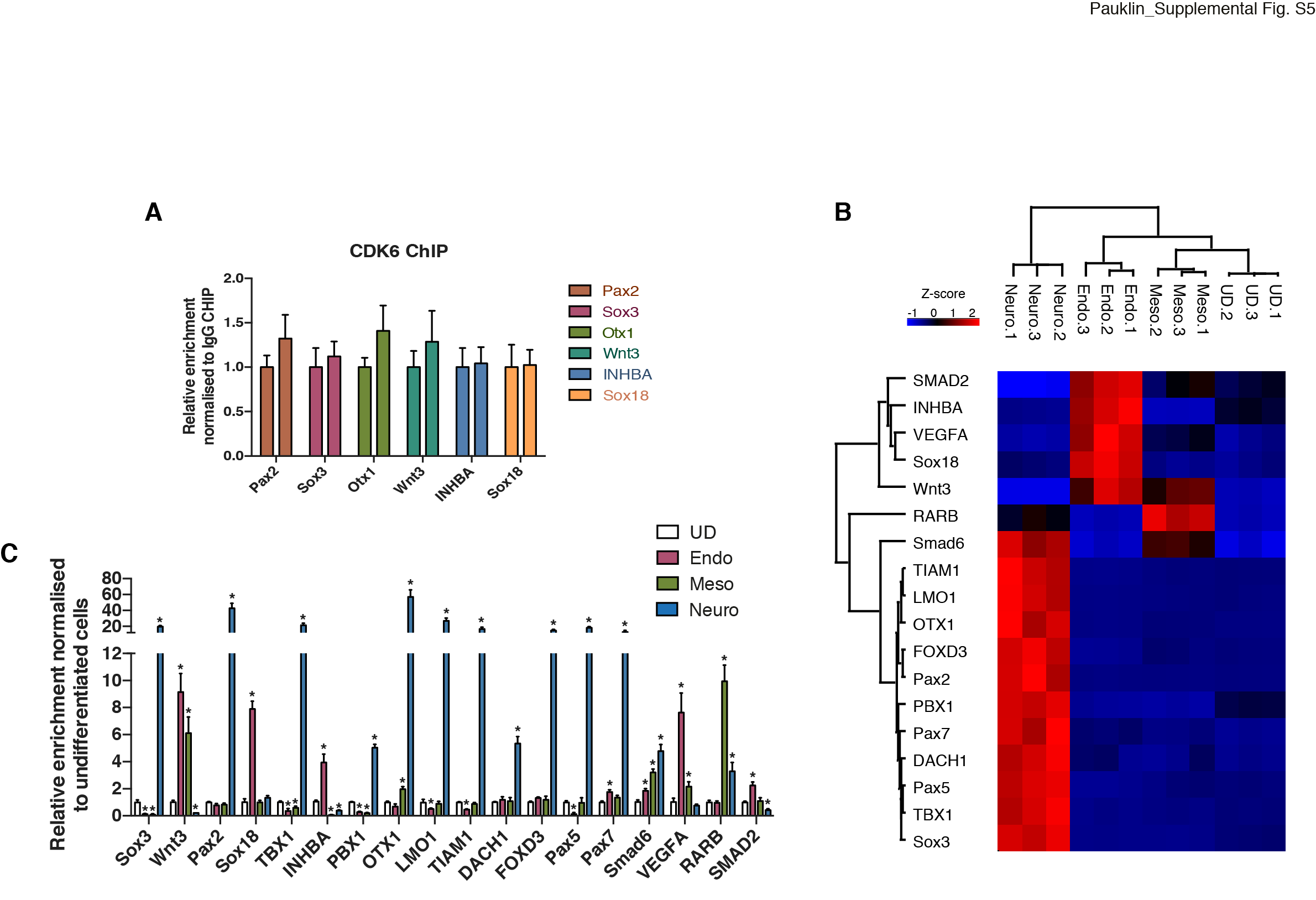

Supplement: Supplemental Material [file supp_30.4.421_Fig_S5.tif]

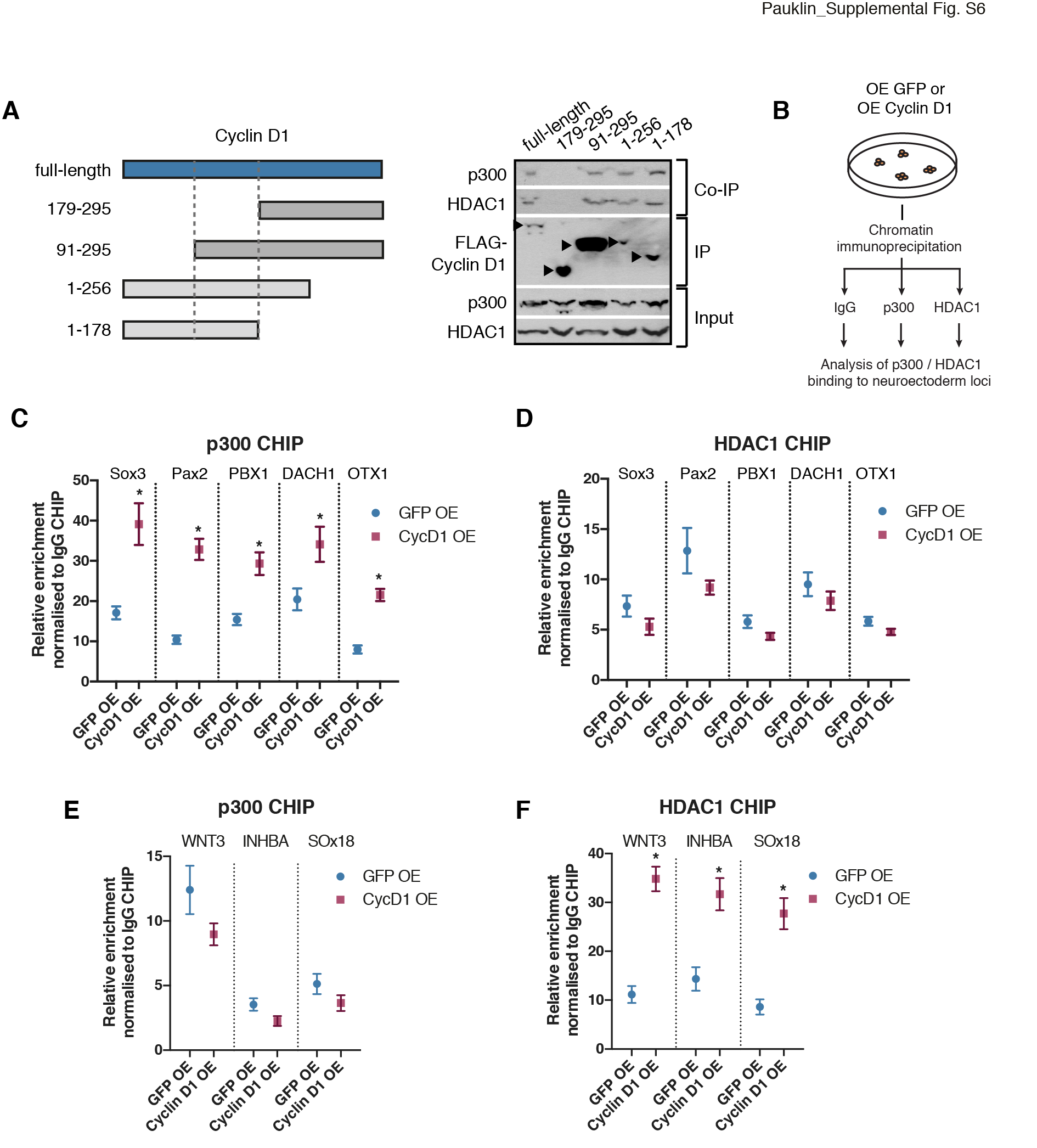

Supplement: Supplemental Material [file supp_30.4.421_Fig_S6.tif]

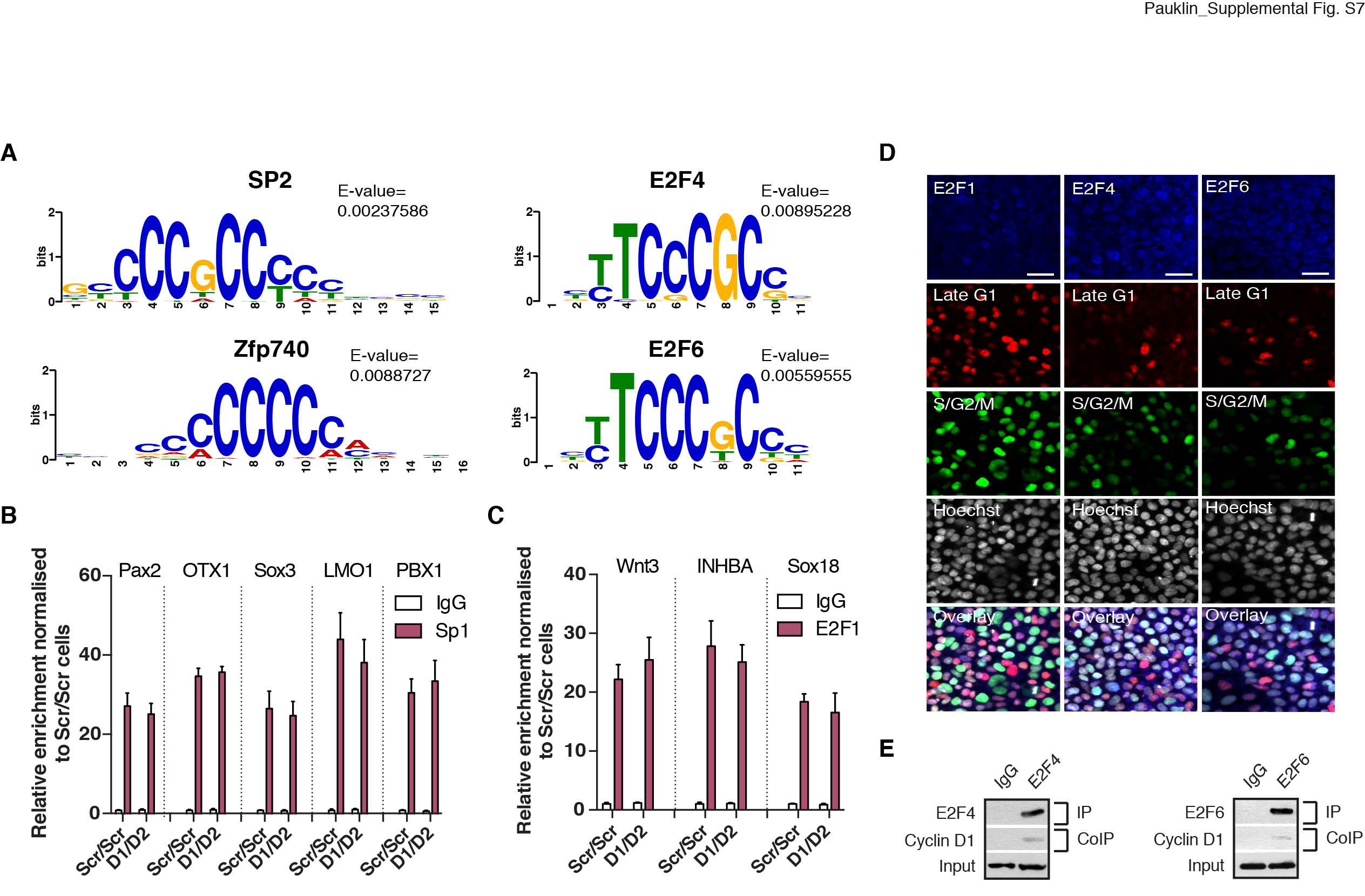

Supplement: Supplemental Material [file supp_30.4.421_Fig_S7.tif]
