# Supplementary material for: Initiation of stem cell differentiation involves cell cycle-dependent regulation of developmental genes by Cyclin D
Source: Genes Dev. 2016 Feb 15;30(4):421–33. doi: 10.1101/gad.271452.115 (PMC4762427; doi:10.1101/gad.271452.115)
Supplement: Supplemental Material [file supp_30.4.421_SuppMaterial.docx]

**Supplemental Information**

**Initiation of stem cell differentiation involves cell cycle-dependent**

**transcription of developmental genes by Cyclin D**

Siim Pauklin^1,^*, Pedro Madrigal^1,2^, Alessandro Bertero^1^, and Ludovic Vallier^1,2,^*

^1^ Wellcome Trust - Medical Research Council Cambridge Stem Cell Institute, Anne McLaren Laboratory for Regenerative medicine and Department of Surgery, University of Cambridge, UK.

^2^ Wellcome Trust Sanger Institute, Hinxton, UK

* Correspondence to: sp579@cam.ac.uk and lv225@cam.ac.uk

**LIST OF CONTENTS:**

**Supplemental Figures (page 4-8)**

- Supplemental Figure 1, related to Figure 1. Nuclear Cyclin D overexpression induces neuroectoderm and blocks endoderm/mesoderm differentiation.
- Supplemental Figure 2, related to Figure 2. Cyclin D1 T286A mutant regulates stem cell differentiation.
- Supplemental Figure 3, related to Figure 2 and Supplementary Tables S1-7. Nuclear Cyclin D regulates differentiation even if CDK4/6 is inhibited in the cells.
- Supplemental Figure 4, related to Figure 3. Cyclin D1 binds to developmental loci in stem cells.
- Supplemental Figure 5, related to Figure 4. Cyclin D1 target genes have distinct expression profiles upon germ layer specification.
- Supplemental Figure 6, related to Figure 5. Cyclin D1 binds directly to p300 and HDAC1 and induces neuroectoderm loci while repressing endoderm loci.
- Supplemental Figure 7, related to Figure 6. Cyclin D1 cooperates with SP1 and E2F1 to regulate the expression of developmental loci.

**Supplemental Tables (online)**

- Supplemental Table S1, related to Figure 4 and Figure S4. List of Cyclin D1 peaks in hESCs, and its associated genes.
- Supplemental Table S2, related to Figure 4 and Figure S4. Cyclin D1 peak distance to gene body.
- Supplemental Table S3, related to Figure 4 and Figure S4. Cyclin D1 peak distance to TSS.
- Supplemental Table S4, related to Figure 4 and Figure S4. Cyclin D1 binds to developmental loci in stem cells.
- Supplemental Table S5, related to Figure 4 and Figure S4. Expression of Cyclin D1 target genes in germ layers.
- Supplemental Table S6, related to Figure 4C. Effects of Cyclin D1 OE or KD on the expression of its target genes.
- Supplemental Table S7, related to Figure 6 and Figure S6. Transcription factor binding motifs identified by MEME-ChIP on the DNA sequences associated to Cyclin D1 peaks.
- Supplemental Table S8-S10, related to Material and Methods and Supplemental Material and Methods. Lists of primers and reagents used for the study.

**Supplemental Material and Methods (pag. 9-28)**

**Supplemental References (pag. 29-31)**

**Supplemental Figure Legends.**

**Figure S1.** Nuclear Cyclin D overexpression induces neuroectoderm and blocks endoderm/mesoderm differentiation. (A) Schematic overview of the approach used to generate Cyclin D1 T286A overexpression (OE) hESC lines. (B) Cyclin D overexpression does not alter the propensity for apoptosis. (C) Histological sections of teratomas derived from (upper panel) GFP or (lower panel) Cyclin D1 overexpressing hESCs. (D) Kinase assay of CDK4/6 activity confirms the specificity of CDK4/6 inhibitor PD0332991. Western blot of pRb in vitro phosphorylation by CDK4 immunoprecipitated from hESCs that were treated with DMSO or PD0332991 for 2h. (E) Cyclin D1 induction during endoderm specification blocks differentiation. Cyclin D1 was transfected into day 1 endoderm cells and analysed by Q-PCR after 24h of antibiotic selection to remove non-transfected cells. Significant differences compared to OE GFP calculated by t-test are marked. (F) Cell cycle profile in Fucci-hESCs upon Cyclin D1 overexpression and CDK4/6 inhibition. Individual dot blot graphs (left) indicate that CDK4/6 inhibition results in the accumulation of cells in late G1 phase, and depicted by bar graps (right). (G-H) CDK4/6 knockdown is not sufficient to fully abolish the neuroectoderm-inducing effects of Cyclin D1. (G) Western blot analysis of CDK4 and CDK6 knockdown by shRNA in hESCs. Two verified shRNA constructs targeting CDK4 or CDK6 specifically reduce their protein expression. (H) OE Cyclin D1 partially maintains its ability to induce neuroectoderm marker expression upon CDK4/6 knockdown. Q-PCR analysis of differentiation markers. Significant differences compared to OE GFP calculated by t-test are marked. (I) Phosphorylation of pRb protein by Cyclin D1 mutants. Western blot analysis of P780-pRb in Cyclin D overexpressing cells.

**Figure S2.** Cyclin D1 T286A mutant regulates stem cell differentiation. (A) Cyclin D cellular location during differentiation. Graphs represent densitometric measurements of relative band intensities. (B) Cyclin D1 is expressed primarily in late G1 phase cells. Immunostaining of Cyclin D1 protein in undifferentiated Fucci-hESCs and endoderm cells. (C) Expression of neuroectoderm markers in Cyclin D1 T286A overexpressing cells shown by Q-PCR. Significant differences compared to OE Control and calculated by t-test are marked. (D) Cyclin D1 T286A overexpression causes neuroectoderm differentiation. Marker expression was analysed in Cyclin D1 T286A mutant cells by Q-PCR. Significant differences compared to OE Control and calculated by t-test are marked. (E-F) Nuclear Cyclin D regulates differentiation. (E) Differentiation marker and (F) pluripotency marker expression was analysed in Cyclin D1 T286A mutant cells by flow cytometry. Significant differences compared to OE PTP6 and calculated by t-test are marked. (G) Cyclin D1-T286A mutant expression induces neuroectoderm differentiation in all cell cycle phases. Cyclin D1-T286A mutant was stably expressed in Fucci-hESCs and sorted into distinct cell cycle phases for Q-PCR analysis of neuroectoderm markers.

**Figure S3.** Nuclear Cyclin D regulates differentiation even if CDK4/6 is inhibited in the cells. (A-F) Cyclin D1 T286A induces neuroectoderm and blocks endoderm differentiation. (A) Immunofluorescence microscopy of pluripotency and differentiation marker expression in Cyclin D1 T286A mutant cells. Cyclin D1 T286A mutant cells differentiated into (B) neuroectoderm, (C) endoderm or (D) mesoderm, and analysed by Q-PCR or western blot for germ layer markers. Significant differences compared to OE GFP and calculated by t-test are marked. Cyclin D1 T286A mutant cells were differentiated into (E) neuroectoderm or (F) endoderm and analysed by immunostaining. Scale bar, 100μm. (G) CDK4/6 inhibition does not block the function of nuclear Cyclin D in regulating neuroectoderm loci in hESCs. CDK4/6 inhibition in CycD1-T286A cells only partially inhibits Cyclin D function. Significant differences calculated by two-way ANOVA are marked. (H) CDK4/6 inhibition is not sufficient to bypass endoderm differentiation in late G1. Sorted Fucci-hESCs were differentiated into endoderm in the presence or absence of 0.75μM PD0332991 and analysed by flow cytometry after 1 or 2 days of endoderm differentiation. Significant differences compared to Day 2 late G1 and calculated by t-test are marked. (I-J) Flag-NLS-Smad2 promotes endoderm differentiation in asynchronous Fucci-hESCs. (I) Flag ChIP of Flag-NLS-Smad2 protein in asynchronous hESCs. Cells were transfected with Flag-NLS-Smad2 expressing construct and analysed by ChIP-Q-PCR on endoderm loci or (J) by Q-PCR of endoderm marker expression. Significant differences compared to untreated Fucci-hESCs (UD) calculated by t-test are marked. Data shown as mean±s.d. (n=3).

**Figure S4.** Cyclin D1 binds to developmental loci in stem cells. (A) Representative Cyclin D1 binding regions spanning 50 kb upstream and 50 kb downstream the binding peak. Cyclin D1 binding was identified by ChIP-sequencing analysis of endogenous Cyclin D1 in hESCs. (B) Global Cyclin D1 target gene expression in ectoderm, endoderm and mesoderm germ layer. (C) Effects of Cyclin D1 target gene overexpression in hESCs. Sox3, PBX1 and Sox18 and GFP expressing plasmids were transfected into H9 hESCs, cultured for 48h before adding puromycin for 6 days and then analysed for neuroectoderm marker (Sox1 and Pax6) and endoderm marker (Sox17 and GSC) expression by Q-PCR. Significant differences compared to OE GFP and calculated by t-test are marked. (D) Cyclin D1 ChIP-Q-PCR at genomic regions close to developmental loci. Significant differences compared to each IgG CHIP sample and calculated by t-test are marked. Data shown as mean±s.d. (n=3).

**Figure S5.** Cyclin D1 target genes have distinct expression profiles upon germ layer specification. (A) CDK6 is not enriched on Cyclin D1 target loci. CDK6 ChIP-Q-PCR on Cycln D1 binding regions of developmental genes. (B-C) Cyclin D1 target gene expression in germ layers. (B) Euclidian hierarchical clustering of gene expression for differentiation and pluripotency markers in H9 cells differentiated for 2 days to endoderm and mesoderm, and 6 days to neuroectoderm. Z-scores in the heat map indicate the differential expression measured in number of standard deviations from the average level across all conditions. (C) The corresponding bar graph of the expression of selected developmental genes identified as Cyclin D1 target loci is also added with normalization to undifferentiated cells. Significant differences compared to undifferentiated H9 (UD) and calculated by t-test are marked. Data shown as mean±s.d. (n=3).

**Figure S6.** Cyclin D1 binds directly to p300 and HDAC1 and induces neuroectoderm loci while repressing endoderm loci. (A) Cyclin D1 truncation constructs map the binding region to p300 and HDAC1. Cyclin D1 truncation constructs were transfected into cells and immunoprecipitated after 48 hours from nuclear extracts. (B-D) Cyclin D1 overexpression increases p300 binding to neuroectoderm loci and HDAC1 to endoderm loci. (B) Schematic overview of analyzing the effects of Cyclin D overexpression of p300 and HDAC1 binding to neuroectoderm loci. (C) P300 and (D) HDAC1 CHIP in OE GFP and OE Cyclin D1 hESCs. Significant differences compared to OE GFP and calculated by t-test are marked. (E-F) Cyclin D1 overexpression increases HDAC1 binding to endoderm loci. (E) P300 and (F) HDAC1 CHIP in OE GFP and OE Cyclin D1 hESCs. Significant differences compared to OE GFP and calculated by t-test are marked. Data shown as mean±s.d. (n=3).

**Figure S7.** Cyclin D1 cooperates with SP1 and E2F1 to regulate the expression of developmental loci. (A) Transcription factor motifs found within Cyclin D1 ChIP-seq peaks. Motif analysis was carried out by MEME-ChIP on DNA sequences associated to Cyclin D1 peaks. Only motifs with E > 0.01 are shown. Full list of identified TF motifs are listed in Supplemental Table S7. (B-C) Cooperation of Cyclin D1 with SP1 and E2F1 on its target loci. Absence of Cyclin D does not affect the binding of (B) SP1 to neuroectoderm loci and (C) E2F1 to endoderm loci. SP1 and E2F1 CHIP was performed in Scr/Scr and Cyclin D1/D2 double knockdown cells. Data shown as mean±s.d. (n=3). (D) Expression of E2F1, E2F4 and E2F6 in hESCs. Immunostaining of E2Fs in Fucci-hESCs. (E) E2F4 and E2F6 form a complex with Cyclin D1 in hESC chromatin fraction. E2F4 and E2F6 were immunoprecipitated from the chromatin fraction of hESCs and analysed by western blotting to detect Cyclin D1 signal.

**Materials and methods.**

**Cell culture of hESCs.**

hESCs (H9 from WiCell) and mEpiSCs were grown in defined culture conditions as described previously ([Brons et al. 2007](#_ENREF_2)). H9 cells were passaged weekly using collagenase IV and maintained in chemically defined medium (CDM) supplemented with Activin A (10 ng/ml) and FGF2 (12 ng/ml).

**Differentiation of hESCs.**

hESCs were differentiated into neuroectoderm, endoderm and mesoderm as described previously ([Vallier et al. 2009](#_ENREF_16)). Briefly, cells were cultured in CDM supplemented with SB-431542 (10 μM; Tocris) and FGF2 (12 ng/ml) for neuroectoderm, in CDM+PVA supplemented with Activin A (100 ng/ml), FGF2 (20 ng/ml), BMP4 (10 ng/ml), Ly294002 (10 μM; Promega) and CHIR99021 (3 μM; Selleck) for mesoderm and in CDM-PVA supplemented with Activin A (100 ng/ml), FGF2 (20 ng/ml), BMP4 (10 ng/ml) and Ly294002 (10 μM; Promega) for endoderm. hESCs were differentiated as described before ([Pauklin and Vallier 2013](#_ENREF_12)).

**Teratoma assays.**

Animal procedures were performed in accordance with the local committee on Animal Experimentation at University of Cambridge. One million hESC were injected in kidney capsule of 6 to 8-weeks-old SCID mice. Three animals were injected in each group. After 12 weeks, mice were sacrificed, and the kidneys and tumours were dissected and fixed for 48h in Bouins solution (Sigma-Aldrich). The fixed tissues were then paraffin-embedded and processed according to standard procedures. Sections (5µm) were stained with hematoxylin/eosin and subsequently examined under bright-field microscope for the presence of tissues deriving from the three germ layers.

**Q-PCR and immunostaining.**

Methods for Q-PCR and immunostaining have been described previously ([Vallier et al. 2009](#_ENREF_16)). Q-PCR data are presented as the mean of three independent experiments and error bars indicate standard deviations. Primer sequences and antibodies have been listed in Supplemental Information.

For immunostaining, cells were fixed for 20 minutes at 4°C in PBS 4% PFA, rinsed three times with PBS, and blocked and permeabilized at the same time using PBS with 10% Donkey Serum (Biorad) and 0.1% Triton X-100 (Sigma) for 30 minutes at room temperature. Overnight incubation at 4°C with the primary antibodies diluted in PBS 1% Donkey Serum 0.1% Triton X-100 was followed by three washes with PBS and further incubation with AlexaFluor secondary antibodies (Invitrogen) for 1 hour at room temperature protected from light. Cells were finally washed three times with PBS, and Hoechst (Sigma) was added to the first wash to stain nuclei. Images were acquired using a LSM 700 confocal microscope (Leica).

**Generating Cyclin D double knockdown cells and CDK4/6 knockdown cells.**

Previously validated shRNA expression vectors (Open Biosystems, Cat no. RHS4533-NM053056, RHS4533-NM001759, RHS4533-NM001136017) directed against Cyclin D1, D2 or D3 were transfected into H9 hESCs with lipofectamine ([Vallier et al. 2004](#_ENREF_15)) and grown for 3 days. Cells were then cultured in the presence of puromycin until antibiotic resistant colonies appeared. These were picked and characterised for knockdown efficiency. For Cyclin D double knockdown, single knockdown sublines were stably transfected with a second shRNA expression vector directed against a different Cyclin D and containing a hygromycin resistance gene. Double knockdown cells were cultured in the presence of puromycin and hygromycin until colonies appeared. These were picked and characterised for knockdown efficiency. For CDK4/6 we used previously validated shRNA expression vectors directed against CDK4 (Sigma, TRCN0000196986 and TRCN0000196698 or CDK6 TRCN0000199114 and TRCN0000196337).

**Generating Cyclin D1 mutant cells.**

cDNA of Cyclin D mutant or truncations was cloned into the pTP6 vector ([Pratt et al. 2000](#_ENREF_13)) with an N-terminal FLAG-HA tag, under the regulation of CAG promoter. The inserts were confirmed by sequencing. Vectors were transfected into H9 hESCs by lipofection ([Vallier et al. 2004](#_ENREF_15)) and grown for 3 days. Thereafter, cells with a stable integration were selected by continuous presence of puromycin. Individual clones were picked, propagated and analysed for subsequent analyses. Alternatively we used transient transfection.

**Flow cytometry.**

Flow cytometry was carried out with a BD MoFlo flow cytometer and analysed by FloJo software. Cell cycle distribution was analysed by Click-It EdU incorporation Kit (Invitrogen) according to manufacturer’s guidelines. Marker expression was analysed at various timepoints during differentiation by first dissociating cells into single cells with Cell Dissociation Buffer (Gibco) and fixing in 4% PFA for 20 min at 4°C. This was followed by permeabilisation and blocking with 10% serum + 0.1% Triton X-100 in PBS for 30 min at RT and incubation with primary antibody in 1% serum + 0.1% Triton X-100 for 2h at 4°C. After washing the samples three times with PBS, they were incubated with a secondary antibody for 2h at 4°C, washed three times with PBS and analysed by flow cytometry.

**Cell sorting by FACS.**

FACS was performed as described before ([Sakaue-Sawano et al. 2008](#_ENREF_14)). In sum, hESCs were washed with PBS and detached from the plate by incubating them for 10 min at 37 °C in Cell Dissociation Buffer (Gibco). Cells were washed with cold PBS and then subjected to FACS with a BeckmanCoulter MoFlo MLS high-speed cell sorter, using parameters described previously ([Sakaue-Sawano et al. 2008](#_ENREF_14)).

**Luciferase assay.**

Genomic regions corresponding to Cyclin D1 binding regions of individual Cyclin D1 target genes were inserted into a pGL3 luciferase construct (Promega) and transfected with Renilla luciferase at a ratio of 10:1, using Lipofectamine 2000 (Invitrogen) ([Vallier et al. 2004](#_ENREF_15)). Luciferase activity was measured with the dual luciferase assay kit following (Promega) manufacturer instructions. Firefly luciferase activity was normalized to Renilla luciferase activity for cell numbers and transfection efficiency. Samples were analysed on a Glomax Luminometer and software.

**Chromatin immunoprecipitation (ChIP).**

hESCs were washed with PBS and detached from the plate by incubating them for 10 min at 37 °C in Cell Dissociation Buffer (Gibco). ChIP was carried out as described before ([Bienvenu et al. 2010](#_ENREF_1); [Casimiro et al. 2012](#_ENREF_3); [Pauklin and Vallier 2013](#_ENREF_12)), except that crosslinking was performed in solution in PBS if samples were sorted by FACS. Data for each cell cycle was normalized to IgG control of each cell cycle phase. We used additional controls as follows: 1) Input of each cell cycle phase and 2) primers for a chromatin region downstream of Smad7 locus known to be negative for histone marks. All of these controls supported our results indicating an enrichment of Cyclin D proteins to neuroectoderm and endoderm loci in late G1 phase. Antibodies for Cyclin D ChIP have been used previously ([Landis et al. 2006](#_ENREF_9)). Two biological replicates and one input DNA as control sample were sequenced at the Cambridge Institute NGS service of the University of Cambridge using Illumina HiSeq, and the raw data have been deposited on ArrayExpress under accession E-MTAB-3807.

**ChIP-seq data analysis.**

Sickle v1.33 (Joshi and Fass, 2011) was applied to raw sequencing data (SE reads, 50 bp) with parameters -q 20 -l 30 . The percentage of reads discarded was very low (~ 1%) for all samples. Reads kept were then aligned to hg38/GRCh38_15 using Bowtie v2.2.2 ([Langmead and Salzberg 2012](#_ENREF_10)), reporting an alignment rate >96.8% for ChIP samples, and 99.12% for the input sample. Mapped reads with a minimum Mapping Quality score of 10 were kept for further processing.

PeakRanger ([Feng et al. 2011](#_ENREF_5)) was used to call peaks in each biological replicate at a False Discory Rate (FDR) ≤ 0.01 (-p 1e-5 -q 0.01).  400 peaks common in both replicates for automosomal and sex chromosomes were selected for further processing.

Using BEDtools ('window -w 50000') we identified 1613 target genes (1519 unique genes) (GENCODE v22) in a region spanning 50 kb  upstream and 50 kb downstream of the final set of peaks, where about half correspond protein-coding genes. 740 protein-coding genes IDs were submitted to Biomart GO Enrichment tool ([http://biomart.org/](http://biomart.org/)), Cut-off *P* ≤ 0.05. 15 GO terms were enriched after multiple hypothesis correction (adjusted *P* ≤ 0.05). ChIP-seq peaks were annotated using the Bioconductor package ChIPpeakAnno ([Zhu et al. 2010](#_ENREF_18)) using the dataset  "hsapiens_gene_ensembl" from Ensembl. Proximal promoter and immediate downstream were  considered 10 kb upstream or 5Kb downstream, respectively, from  the transcription start sites. 400 DNA sequences associated to the peaks were submitted to MEME-ChIP ([Machanick and Bailey 2011](#_ENREF_11)) for motif analysis (motif database: "Vertebrates (in vivo and in silico", expected motif site distribution = ANR (Any Number of Repetitions), MEME search for n=20 motifs, maximum width = 20 bp, rest of parameters default).

740 protein-coding genes were associated to FPKM values and differential expression analysis of RNA-seq (HUES64 vs Endoderm, HUES64 vs Mesoderm, or HUES64 vs Ectoderm) for all Protein-coding genes expressed in hESCs and the differentiated populations (Gifford et al., 2013). Comparison to Cyclin D1-bound genomic regions identified in Cyclin D1 ChIP-chip was performed as follows: peaks were translated from mm7 (26284) to hg18, then hg18 to hg38 using LiftOver, resulting in 14330 lifted regions in hg38). Out of 400 peaks in hESC, 14 overlapping binding sites were identified. Visualisation of Cyclin D1 binding in figures correspond to reproducible peaks in one of the replicates.

**Sequential chromatin immunoprecipitation.**

For sequential CHIP, the first CHIP was performed as described above. The elutions of first CHIP were used for the second CHIP by adding 5.2ml of dilution buffer to each sample and then continued with primary antibody incubation according to the usual CHIP protocol described above.

**Cell fractionations.**

Cells were harvested with trypsin and washed twice with cold PBS. For cytoplasmic lysis, cells were suspended in 5 times packed cell volume (1 ul PCV = 10^6^ cells) equivalent of Isotonic Lysis Buffer (10 mM Tris HCl, pH 7.5, 3 mM CaCl, 2 mM MgCl_2_, 0.32 M Sucrose, Complete protease inhibitors and phosphatase inhibitors), and incubated for 12 min on ice. Triton X-100 was added to a final concentration of 0.3% and incubated for 3 min. The suspension was centrifuged for 5 min at 1,500 rpm at 4 °C and the supernatant (cytoplasmic fraction) transferred to a fresh chilled tube. For nuclear lysis, nuclear pellets were resuspended in 2 x PCV Nuclear Lysis Buffer+Triton X-100 (50 mM Tris HCl, pH 7.5, 100 mM NaCl, 50 mM KCl, 2 mM MgCl_2,_ 1 mM EDTA, 10% Glycerol, 0.3% Triton X-100, Complete protease inhibitors and phosphatase inhibitors) and dounce homogenized. The samples were incubated with gentle agitation for 30 min at 4 °C and then centrifuged with a Ti 70.1 rotor at 22,000 rpm for 30 min at 4 °C or with a Ti 45 rotor for 30 min at 20,000 rpm at 4 °C. The chromatin pellets were dounce homogenized in 2 x PCV Nuclear Lysis Buffer+Triton X-100 and Benzonase until the pellets gave much less resistance. The samples were incubated at RT for 30 min and centrifuged with either a Ti 70.1 rotor for 30 min at 22,000 rpm at 4 °C or with a Ti 45 rotor for 30 min at 20,000 rpm at 4 °C.

**Protein co-immunoprecipitation.**

Antibodies were cross-linked to Protein G-Agarose beads (Roche, 1 ug of antibody per 5 ul of beads) with dimethyl pimelimidate (Sigma) using standard biochemical techniques, prior to performing immunoprecipitations. Samples were incubated with 5 ug of cross-linked antibodies for 12h at 4 °C. Beads were washed five times with ten bead volumes of Nuclear Lysis Buffer and eluted in SDS western blotting buffer (30 mM Tris pH 6.8, 10% Glycerol, 2% SDS, 0.36 M beta-mercaptoethanol (Sigma), 0.02% bromophenol blue) by heating at 90 °C for 5 min. Samples were analysed by standard western blotting techniques.

**Interaction studies of Cyclin D truncations with p300 and HDAC1.**

Cyclin D mutants and truncation constructs (Supplemental File 1D and ([Kim et al. 2000](#_ENREF_8); [Fu et al. 2005](#_ENREF_6)) were transfected into hESCs. 48 hours after transfection, Cyclin D was immunoprecipitated by FLAG antibody as described above and samples were analysed for the efficiency of p300 and HDAC1 co-immunoprecipitation.

**Transfection with constitutively activated Activin receptor.**

The Activin receptor and its mutant have been described before ([Wang et al. 1996](#_ENREF_17)).

**Overexpression of Cyclin D1 target genes.**

TetO-FUW-SOX3 was a gift from Rudolf Jaenisch (Addgene plasmid # 61546) ([Cassady et al. 2014](#_ENREF_4)), PBX1A-pCMV1 was a gift from Corey Largman (Addgene plasmid # 21029), pCMV5 Smad2-HA was a gift from Joan Massague (Addgene plasmid # 14930) ([Hata et al. 1997](#_ENREF_7)). The coding region of human Sox18 was cloned into pTP6 plasmid with EcoRI and XhoI by using the following Forward and Reverse primers: 5’ NNNGAATTCATGCAGAGATCGCCGCCCGGC and NNNCTCGAGGGCATTTTAAAAATTTATTTACAAAGTTGTGTACAACACGATGG. hESCs were transcfected with Lipofectamine2000 (Invitrogen) and puromycin selection was started 48h after transfection.

**Kinase assays.**

hESCs were cultured with or without CDK inhibitor for 2h, then washed with cold PBS, and collected in cold CDB with protease and phosphatase inhibitors. After pelleting, cells were washed once again with PBS with protease and phosphatase inhibitors. Cell lysis was performed in D-IP kinase buffer (50mM Hepes ph7.5, 150 mM NaCl, 1mM EDTA, 2.5mM EGTA, 0.1% Tween 20, 10% Glycerol) with protease and phosphatase inhibitors using 5x cell pellet volume, and incubated on rotating wheel for 30min. Lysates were then clarified by centrifuging at max speed at 4°C for 15min, and quantified for protein concentration. 500ug were incubated with 5ug of anti-CDK4/6 Ab for 2h at 4°C rotating. Antibody was captured with 40ul of pre-washed protein G-agarose beads (50%-50%) for 1h at 4°C by rotation. Beads were washed 3x in 1ml D-IP kinase buffer with protease and phosphatase inhibitors and then 3x with 1ml of kinase reaction buffer (50mM Hepes ph7.2, 10mM MgCl2, 5mM MnCl2, 1mM DTT). Liquid was aspirated from beads using and then resuspended into 30ul kinase reaction mix (1x kinase reaction buffer, 200uM ATP, 2ug Rb-C term protein). For the negative control, the same buffer without ATP was used, while for the positive control the same buffer was added to recombinant CDK4/CyclinD1 (400ng corresponding to 4ul). As an additional control, the same reaction was prepared with the CDK inhibitor at a 5x less concentration than used on the cells. The reactions were incubated at 37°C with gentle shaking for different time points: 15min, 30min, 1h, 2h. The controls were kept for longer time points. The samples were then boiled for 5min 95°C in SDS western blot running buffer before freezing. Samples were run on a precast 4-12% gel by loading 1/2 of the kinase assay.

**Statistical analysis.**

GraphPad Prism 5 was used for statistical analysis. t-test and two-way ANOVA tests followed by Bonferroni’s corrected multiple comparisons between pairs of conditions. Unless otherwise indicated in the figure legends, we analysed three biological replicates for each data point in all graphs. P≤0.05 marked with (*).

**Supplemental Table S8. Q-PCR primers.**

| Gene | Primer name | Sequence |
| --- | --- | --- |
| PBGD | PBGDF | GGAGCCATGTCTGGTAACGG |
|  | PBGDR | CCACGCGAATCACTCTCATCT |
| Oct4 | Pou5F_F | AGTGAGAGGCAACCTGGAGA |
|  | Pou5F_R | ACACTCGGACCACATCCTTC |
| Nanog | NanogF | CATGAGTGTGGATCCAGCTTG |
|  | NanogR | CCTGAATAAGCAGATCCATGG |
| Sox2 | hSox2F | TGGACAGTTACGCGCACAT |
|  | hSox2R | CGAGTAGGACATGCTGTAGGT |
| Nestin | NestinF | GAAACAGCCATAGAGGGCAAA |
|  | NestinR | TGGTTTTCCAGAGTCTTCAGTGA |
| Pax6 | hPax6F | CTTTGCTTGGGAAATCCGAG |
|  | hPax6R | AGCCAGGTTGCGAAGAACTC |
| Sox1 | Sox1Q | QUANTITECT PRIMERS (QIAGEN) |
| GSC | DAGSCF | GAGGAGAAAGTGGAGGTCTGGTT |
|  | DAGSCR | CTCTGATGAGGACCGCTTCTG |
| Brachury | DABraF | TGCTTCCCTGAGACCCAGTT |
|  | DABraR | GATCACTTCTTTCCTTTGCATCAAG |
| Eomes | hEomesF | ATCATTACGAAACAGGGCAGGC |
|  | hEomesR | CGGGGTTGGTATTTGTGTAAGG |
| Sox17 | hsox17F | CGCACGGAATTTGAACAGTA |
|  | hsox17R | GGATCAGGGACCTGTCACAC |
| MixL1 | hMixL1F | GGTACCCCGACATCCACTTG |
|  | hMixL1R | TAATCTCCGGCCTAGCCAAA |
| Mesp1 | Mesp1F | GAAGTGGTTCCTTGGCAGAC |
|  | Mesp1R | TCCTGCTTGCCTCAAAGTGT |
| Mesp2 | Mesp2F | AGCTTGGGTGCCTCCTTATT |
|  | Mesp2R | TGCTTCCCTGAAAGACATCA |
| Hoxb1 | Hoxb1F | AGAACCGACGAATGAAGCAG |
|  | Hoxb1R | GACTGGTCTGAGGCATCTCC |
| CCND1 | CCND1F | GCCGTCCATGCGGAAGATC |
|  | CCND1R | CCTCCTCCTCGCACTTCTGT |
| CCND2 | CCND2F | TGAGGCGGTGTAGGACAGG |
|  | CCND2R | ATATCCCGCACGTCTGTAGG |
| CCND3 | CCND3F | CATGTACCCGCCATCCAT |
|  | CCND3R | AGCTTCGATCTGCTCCTGAC |
| Sox3 | Sox3F | GGTACAGACCAGGACCGTGT |
|  | Sox3R | GTCGGTCAGCAGTTTCCAGT |
| PBX1 | PBX1F | CAACTCGGCTGGTGGATACC |
|  | PBX1R | GGGAGGTCACTGATGAAGGG |
| MEIS3 | MEIS3F | ATGGCCCGGAGGTATGATGA |
|  | MEIS3R | GCTGTCCAAGCCTGGGG |
| TBX1 | TBX1F | ACGGAGAAAGGGCTGGTCAC |
|  | TBX1R | CCTGACTTGGGCTCTGAAACC |
| Sox18 | Sox18F | TCAGCAAGATGCTGGGCAAA |
|  | Sox18R | CCTTGCGCGCCTGCTTC |
| Pax5 | Pax5F | CCACACCCAAAGTGGTGGAA |
|  | Pax5R | TCCGGATGATCCTGTTGATGG |
| Pax2 | Pax2F | CCCCGCCTTACTAAGTTCCC |
|  | Pax2R | AGACGGGGACGATGTGGA |
| Ngn2 | Ngn2F | GGAGCTGCGCCACAGTAG |
|  | Ngn2R | TTTGACGAACATCTTAGTTGGCT |
| Ngn1 | Ngn1F | GCTCTGAGCGCCTTTCTATCT |
|  | Ngn1R | GTCTGGCACAGTCTTCCTCG |
| Wnt3 | Wnt3F | GGGCCGCACGACTATCC |
|  | Wnt3R | TCCGAGGCGCTGTCATACTT |
| INHBA | INHBAF | ACGTTTGCCGAGTCAGGAAC |
|  | INHBAR | GCGGATGGTGACTTTGGTCC |
| DACH1 | DACH1F | CCACCAACAGATGAGACCCC |
|  | DACH1R | CAGAAGAGTCTCGATGGAAGACAG |
| SMAD6 | SMAD6F | TGAATTCTCAGACGCCAGCA |
|  | SMAD6R | CTGCCCTGAGGTAGGTCGTA |
| Sox18 | Sox18F | TCAGCAAGATGCTGGGCAAA |
|  | Sox18R | CCTTGCGCGCCTGCTTC |
| TWIST2 | TWIST2F | CGCCAGGGCTGTCCGTC |
|  | TWIST2R | TCTTCTGTCCGATGTCACTGCTG |
| HNF4a | HNF4aF | GGCAATGACACGTCCCCATC |
|  | HNF4aR | CCTCCGGAAGAAGCCCTTG |

**Supplemental Table S9. CHIP primers.**

| Sox3 | Sox3F | GCACCAAATTCCACGGCTT |
| --- | --- | --- |
|  | Sox3R | GGCAGGGGGTTGGGTAGAG |
| PBX1 | PBX1F | GGGGTGTATAACTGCCTTCCC |
|  | PBX1R | AGCTCTTGACACCTAGCACC |
| MEIS3 | MEIS3F | TCGATTTGTGGCGATTTGGAG |
|  | MEIS3R | TTTACGGCTGAGGAGGCAAC |
| TBX1 | TBX1F | GTTGCAGTGTAGACAGCCCG |
|  | TBX1R | TGGGCACAAATAAATGGACACG |
| Sox18 | Sox18F | GAGGTCAGAACTGGGGTCCA |
|  | Sox18R | GCAACAGCCCAGAAAGAGTAA |
| Pax5 | Pax5F | TGGGGGCTTTAGGGATCTGG |
|  | Pax5R | GAGGCGACGTTCCGGTTTA |
| Pax2 | Pax2F | CAGTCTTCAGCCCGACATCA |
|  | Pax2R | ATGGAAAGGAGGGCTTAAGGA |
| Ngn2 | Ngn2F | TGATAACATCTAATTTCACGGAGCG |
|  | Ngn2R | GACTGCCCACAAATCAACCG |
| Ngn1 | Ngn1F | TGGAGGTCTTCAAGAAAGCACTA |
|  | Ngn1R | AACTTGGAAAGCTCTTGGCTGA |
| Wnt3 | Wnt3F | CGACCCCTTCCTCGCCT |
|  | Wnt3R | GCGCATAGTAGGTAAGCACCA |
| INHBA | INHBAF | CTGACAGCACTGCCCTCTAC |
|  | INHBAR | TGATTGAGCATGGTGGGAATTG |
| DACH1 | DACH1F | CTCTGGAAGGGAAGTCTGCG |
|  | DACH1R | ACTGGCTACAAAAGCCGCTC |
| SMAD6 | SMAD6F | CCAGAACTCAGAAGGATGGGAG |
|  | SMAD6R | TATGGTAGGTATTGGTGGCCC |
| Sox18 | Sox18F | AGGTCAGAACTGGGGTCCAGA |
|  | Sox18R | AGCAACAGCCCAGAAAGAGTA |
| TWIST2 | TWIST2F | GCTCACAGACTGAAAGGCTGG |
|  | TWIST2R | CCAATGCAGGCACACGAAC |
| HNF4a | HNF4aF | TGCCACTGACCAGTGTGAAC |
|  | HNF4aR | TGGGATTTCTGGGCACTGG |
| HoxD | HoxDF | GTTCCCTTCGGCCTCCC |
|  | HoxDR | AGATAACTACGTCGCCACCG |
| Pax6 | Pax6F | TCGGAGGAGGGGACAGGGTGAT |
|  | Pax6R | GCCGCCGTCCTGTATGGCGTA |
| Sox1 | Sox1F | GAGCTGCAACTTGGCCACGACT |
|  | Sox1R | GTTCGGCTCGCAGGTGGAAAGT |
| Sox2 | Sox2F | CCGCACCTTAGCTGCTTCCCG |
|  | Sox2R | CAACAGGTCACACCACACGCCT |
| Sox17 | Sox17F | GCTTTTCGAGTCTCCCTAACCCCG |
|  | Sox17R | CGAGTCCCACGTCCCAGTCCA |
| EOMES | EOMESF | CTTCTTCCAGCGTGTGAGCCTGG |
|  | EOMESR | CCTGAATCCAGCGTCCTTTCCGG |
| CDX2 | CDX2F | AAGTGCACCAGGTTGGAAGGAGGA |
|  | CDX2R | CAGCGCGCACTCTACGCACA |
| Sox2 | Sox2F.2 | AACATGATGGAGACGGAGCTG |
|  | Sox2R.2 | GGACCACACCATGAAGGCAT |
| Sox2 | Sox2F.3 | ACATGATGGAGACGGAGCTG |
|  | Sox2R.3 | GGGACCACACCATGAAGGC |
| Sox17 | Sox17F.2 | GGGCATCTCAGTGCCTCAC |
|  | Sox17R.2 | TCTGGGTCTGGCTCTGGTC |
| Sox17 | Sox17F.3 | GGACCGCACGGAATTTGAAC |
|  | Sox17R.3 | TAATATACCGCGGAGCTGGC |
| Neg Control | Smad7-veF | accctgataggaagaggggaag |
|  | Smad7-veR | tcacacacactcctgacaagtga |

**Supplemental Table S10. Antibodies.**

|  |  |  |  |
| --- | --- | --- | --- |
| **Antibody raised against** | **Catalog number** | **Company** | **Suitable Application** |
| Actin, clone C4 | MAB1501 | Chemicon |  |
| Brachyury (T) | af2085 | R&D Systems |  |
| Cdx2 | MU392A-UC | BioGenex |  |
| Cyclin D1 (H-295) | sc-753 | Santa Cruz | CHIP total Cyclin D |
| Cyclin D1 (M-20) | sc-718 | Santa Cruz | CHIP D1>D2 |
| Cyclin D1 (C-20) | sc-717 | Santa Cruz | WB |
| Cyclin D1 (DCS-6) | sc-20044 | Santa Cruz | WB |
| Cyclin D1 (HD11) | sc-246 | Santa Cruz | WB |
| Cyclin D2 (C-17) | sc-181 | Santa Cruz | CHIP D2>D1 |
| Cyclin D2 (DCS-3) | sc-56305 | Santa Cruz | WB |
| Cyclin D2 (DCS-5) | sc-53637 | Santa Cruz | WB |
| Cyclin D3 (C-16) | sc-182 | Santa Cruz | CHIP D3 |
| Cyclin D3 (D-7) | sc-6283 | Santa Cruz | WB |
| Cyclin D3 (DCS-22) | Ab28283 | Abcam | WB |
| Cyclin D3 (H-292) | sc-755 | Santa Cruz | WB |
| CDK4 (H-22) | sc-601 | Santa Cruz |  |
| CDK6 (C-21) | sc-177 | Santa Cruz |  |
| EOMES | ab23345 | R&D Systems |  |
| Gata4 (G-4) | sc-25310 | Santa Cruz |  |
| HDAC1, clone 2E10 | 05-100 | Millipore |  |
| Histone H3 | ab1791 | Abcam |  |
| Histone H3 (tri methyl K4) | ab8580 | Abcam |  |
| Histone H3 (tri methyl K27) | ab6002 | Abcam |  |
| Nanog | af1997 | R&D Systems |  |
| Nestin (Rat-401) | sc-33677 | Santa Cruz |  |
| Oct-3/4 (C-10) | sc-5279 | Santa Cruz |  |
| Pax6 | PRB-278P-100 | Covance |  |
| P300 | sc-584 | Santa Cruz |  |
| Smad2/3 | AF3797 | R&D Systems |  |
| Sox1 | AF3369 | R&D Systems |  |
| Sox17 | AF1924 | R&D Systems |  |
| Sox2 | AF2018 | R&D Systems |  |
| CXCR4 | MAB173 | R&D Systems |  |
| Tra-1-60 | sc-21705 | Santa Cruz |  |
| Alexa Fluor 647 donkey α-mouse | A31571 | Invitrogen |  |
| Alexa Fluor 647 donkey α-goat | A21447 | Invitrogen |  |
| Sp1 (PEP 2) X | sc-59 X | Santa Cruz |  |
| E2F1 | sc-193 | Santa Cruz |  |
| E2F4 | sc-1082x | Santa Cruz |  |
| E2F6 | sc-8366 | Santa Cruz |  |

**Supplemental Table S11. Cyclin D truncation constructs.**

| **Gene** | **Plasmid name** |
| --- | --- |
| Cyclin D1 | Cyclin D1 wt |
| Cyclin D1 | LxCxE |
| Cyclin D1 | LxGxE Mut (C7G) |
| Cyclin D1 | K112E |
| Cyclin D1 | 1-178 |
| Cyclin D1 | 1-256 |
| Cyclin D1 | 48-295 |
| Cyclin D1 | 91-295 |
| Cyclin D1 | 143-295 |
| Cyclin D1 | 179-295 |

**Supplemental References.**

Bienvenu F, Jirawatnotai S, Elias JE, Meyer CA, Mizeracka K, Marson A, Frampton GM, Cole MF, Odom DT, Odajima J et al. 2010. Transcriptional role of cyclin D1 in development revealed by a genetic-proteomic screen. *Nature* **463**: 374-378.

Brons IG, Smithers LE, Trotter MW, Rugg-Gunn P, Sun B, Chuva de Sousa Lopes SM, Howlett SK, Clarkson A, Ahrlund-Richter L, Pedersen RA et al. 2007. Derivation of pluripotent epiblast stem cells from mammalian embryos. *Nature* **448**: 191-195.

Casimiro MC, Crosariol M, Loro E, Ertel A, Yu Z, Dampier W, Saria EA, Papanikolaou A, Stanek TJ, Li Z et al. 2012. ChIP sequencing of cyclin D1 reveals a transcriptional role in chromosomal instability in mice. *J Clin Invest* **122**: 833-843.

Cassady JP, D'Alessio AC, Sarkar S, Dani VS, Fan ZP, Ganz K, Roessler R, Sur M, Young RA, Jaenisch R. 2014. Direct lineage conversion of adult mouse liver cells and B lymphocytes to neural stem cells. *Stem cell reports* **3**: 948-956.

Feng X, Grossman R, Stein L. 2011. PeakRanger: a cloud-enabled peak caller for ChIP-seq data. *BMC Bioinformatics* **12**: 139.

Fu M, Wang C, Rao M, Wu X, Bouras T, Zhang X, Li Z, Jiao X, Yang J, Li A et al. 2005. Cyclin D1 represses p300 transactivation through a cyclin-dependent kinase-independent mechanism. *J Biol Chem* **280**: 29728-29742.

Hata A, Lo RS, Wotton D, Lagna G, Massague J. 1997. Mutations increasing autoinhibition inactivate tumour suppressors Smad2 and Smad4. *Nature* **388**: 82-87.

Kim HA, Pomeroy SL, Whoriskey W, Pawlitzky I, Benowitz LI, Sicinski P, Stiles CD, Roberts TM. 2000. A developmentally regulated switch directs regenerative growth of Schwann cells through cyclin D1. *Neuron* **26**: 405-416.

Landis MW, Pawlyk BS, Li T, Sicinski P, Hinds PW. 2006. Cyclin D1-dependent kinase activity in murine development and mammary tumorigenesis. *Cancer Cell* **9**: 13-22.

Langmead B, Salzberg SL. 2012. Fast gapped-read alignment with Bowtie 2. *Nat Methods* **9**: 357-359.

Machanick P, Bailey TL. 2011. MEME-ChIP: motif analysis of large DNA datasets. *Bioinformatics* **27**: 1696-1697.

Pauklin S, Vallier L. 2013. The cell-cycle state of stem cells determines cell fate propensity. *Cell* **155**: 135-147.

Pratt T, Sharp L, Nichols J, Price DJ, Mason JO. 2000. Embryonic stem cells and transgenic mice ubiquitously expressing a tau-tagged green fluorescent protein. *Dev Biol* **228**: 19-28.

Sakaue-Sawano A, Kurokawa H, Morimura T, Hanyu A, Hama H, Osawa H, Kashiwagi S, Fukami K, Miyata T, Miyoshi H et al. 2008. Visualizing spatiotemporal dynamics of multicellular cell-cycle progression. *Cell* **132**: 487-498.

Vallier L, Rugg-Gunn PJ, Bouhon IA, Andersson FK, Sadler AJ, Pedersen RA. 2004. Enhancing and diminishing gene function in human embryonic stem cells. *Stem Cells* **22**: 2-11.

Vallier L, Touboul T, Chng Z, Brimpari M, Hannan N, Millan E, Smithers LE, Trotter M, Rugg-Gunn P, Weber A et al. 2009. Early cell fate decisions of human embryonic stem cells and mouse epiblast stem cells are controlled by the same signalling pathways. *PLoS One* **4**: e6082.

Wang Z, Sicinski P, Weinberg RA, Zhang Y, Ravid K. 1996. Characterization of the mouse cyclin D3 gene: exon/intron organization and promoter activity. *Genomics* **35**: 156-163.

Zhu LJ, Gazin C, Lawson ND, Pages H, Lin SM, Lapointe DS, Green MR. 2010. ChIPpeakAnno: a Bioconductor package to annotate ChIP-seq and ChIP-chip data. *BMC Bioinformatics* **11**: 237.
